# A Thermodynamically Consistent, Microscopically-Based, Model of the Rheology of Aggregating Particles Suspensions

**DOI:** 10.3390/e24050717

**Published:** 2022-05-17

**Authors:** Soham Jariwala, Norman J. Wagner, Antony N. Beris

**Affiliations:** Center for Research in Soft Matter and Polymers (CRiSP), Department of Chemical and Biomolecular Engineering, University of Delaware, Newark, DE 19716, USA; sdj@udel.edu (S.J.); wagnernj@udel.edu (N.J.W.)

**Keywords:** thixotropy, viscoelasticity, shear thinning, population balance, aggregation, breakage, TEVP

## Abstract

In this work, we outline the development of a thermodynamically consistent microscopic model for a suspension of aggregating particles under arbitrary, inertia-less deformation. As a proof-of-concept, we show how the combination of a simplified population-balance-based description of the aggregating particle microstructure along with the use of the single-generator bracket description of nonequilibrium thermodynamics, which leads naturally to the formulation of the model equations. Notable elements of the model are a lognormal distribution for the aggregate size population, a population balance-based model of the aggregation and breakup processes and a conformation tensor-based viscoelastic description of the elastic network of the particle aggregates. The resulting example model is evaluated in steady and transient shear forces and elongational flows and shown to offer predictions that are consistent with observed rheological behavior of typical systems of aggregating particles. Additionally, an expression for the total entropy production is also provided that allows one to judge the thermodynamic consistency and to evaluate the importance of the various dissipative phenomena involved in given flow processes.

## 1. Introduction

**Figure 1:**
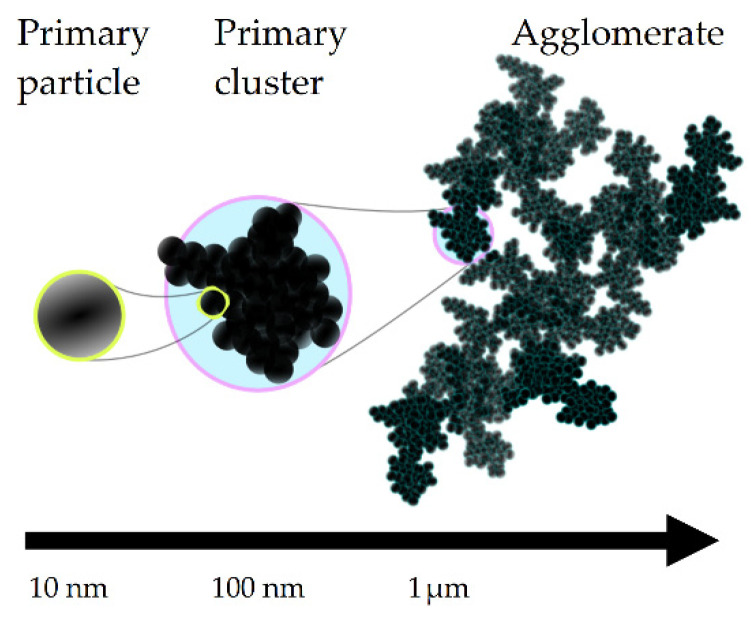
Schematic indicating the spans of length scales involved in fractal agglomerates. Such structures are commonly observed in dispersions such as carbon black in mineral oil [1,27] and fumed silica in paraffin oil [28,29].

Unstable colloidal suspensions exhibit aggregation due to interparticle attraction, which plays a key role in their complex physical properties. This aggregation often results in the formation of fractal agglomerates than can span several orders of magnitude in terms of length scale [1,2,3] (illustrated in Figure 1) and which undergo restructuring when subjected to flow [2,3,4]. This evolution of the structure at mesoscopic length scales manifests macroscopically as time- and structure-dependent flow behavior [2,3]. The material may develop a yield stress, requiring a finite stress to initiate flow. The elastic modulus and viscosity, which are functionals of the extent of the particle network and size distribution [5,6,7], may also change with time when the material is flowing, a phenomenon known in the literature as thixotropy [8,9,10,11,12,13,14].

Historically, a variety of models have been developed [3,12,13,15,16,17,18,19] to describe the rheology of such materials and their flow dynamics. These models have the common feature of connecting the time evolution of structural variables with rheological properties of interest. The most prominent are models based on viscosity [20] and structural parameters [21,22] (many are also summarized in reviews [10,13]). In the viscosity models, a direct connection to a system constituent (such as hematocrit for blood) is established for a key rheological parameter, in this case the viscosity [20]. In the structure-based modeling approach, the structure is characterized by a scalar structural parameter, usually bound between 0 and 1, with unstructured and fully structured states as the extremes. A connection between the rheology and material flow history is then subsequently established by coupling rheological variables to the structural parameter [21,22]. As originally envisioned, the structural parameter is proposed to represent the degree of physical bond formation between agglomerates, analogous to the chemical bond formation during a reaction [11,23]. The evolution equation is, thus, aptly named structural kinetics. The aggregation and breakage of structures under shear forces have been the focus of studies in the context of population balance. For reference, see works from Spicer and Pratsinis, where the authors examine the aggregation and breakage kinetics [24] and the emergent size distribution [25]. However, with the possible exception of the recent work by Mwasame and co-workers [2,3] who proposed a mesoscale population balance model to connect the aggregation dynamics with thixotropy and yield stress, and Jamali et al. [26] who described the microstructural changes in shear flow using a fabric tensor, it has been difficult to make a clear association between the macroscopic stress and physically observable features in structures.

Several models, following the work of de Souza Mendes [30,31], attempt to combine the viscoelasticity, thixotropy and yielding phenomena under the umbrella of thixotropic elasto-viscoplastic (TEVP) behavior to connect their origin to the underlying fluid structure. Dimitriou and McKinley [32] introduced the concept of isotropic hardening (IH) and kinematic hardening (KH) to TEVP modeling to address viscoplasticity in a more fundamental manner by adapting concepts from the plasticity literature, such as back stress, as well as the decomposition of strain into reversible and irreversible components. Kinematic hardening has been customarily used to describe the stress arising from structural formations in structural parameter models [17,33]. In a recent study by Dimitriou and McKinley [34] and Varchanis et al. [35], these advancements were extended to tensorial descriptions of TEVP materials that are valid for general flows exhibiting both extensional and shear characteristics. In parallel, elastic-hardening-based structural thixotropic models have also been developed and successfully used to describe the thixotropic characteristics of blood rheology [36,37]. Blood, another example of an aggregating colloidal system, has long been known to possess a non-Newtonian rheology exhibiting thixotropic as well as viscoelastic characteristics (see recent reviews [38,39]). Two most recent developments in thixotropic modeling relevant to the present work include the development of a population-balance-based approach [40] and tensorial forms of previous structural kinetics models [41].

Despite the success of the structural kinetics modeling approach in terms of prediction and ease of implementation, there remain significant areas for improvement. The associated governing equations are purely phenomenological and constructed to satisfy the rheometrically observed transient and steady-state shear flow features without a direct connection to an experimentally identifiable internal microstructure. In addition, recent studies have called into question the thermodynamic consistency of structural kinetics models. Larson [12] has pointed out the thermodynamic infeasibility of the existence of purely elastic thixotropic materials (also known as thixo-elastic materials). Larson argues that because the breakage term in the structure kinetic equation depends on the total rate of strain, there is a possibility of thixo-elastic materials violating the second law of thermodynamics. If a thixo-elastic system can operate in a closed thermodynamic cycle, the stiffening of such a material (increasing elastic modulus) with time means that the amount of work recovered in the reverse process can exceed the amount of work performed on the system in the forward process due to the time-varying structure and associated rheological properties. This problem was recently analyzed in detail by Joshi [19], whereby the author suggested a resolution of this violation by use of an alternative ‘viscous strain rate’ form of structural kinetics instead of the more widely used ‘total strain rate’ form.

Alternatively, Stephanou and Georgiou [42] have offered an approach to guarantee thermodynamic consistency in structural thixotropic models by generating the governing equations directly from the Hamiltonian function (Helmholtz free energy functional of the system) using the single-generator bracket formulation of nonequilibrium thermodynamics (SGBF-NET) [43]. Although these authors still employ a phenomenological structural kinetics model, their work brings forward some key new developments, including a thermodynamically consistent presentation of the structural kinetics equations and a unified framework for viscoelasticity and viscoplasticity expressed in terms of another structural parameter cast as a conformation tensor density [42]. The SGBF-NET theory, originally developed by Beris and Edwards [43,44,45], has the benefit of allowing one to check the thermodynamic consistency of the governing equations via its formula. It represents one of several recently developed formulations of nonequilibrium thermodynamics [46,47,48,49,50], with the main advantages being the simplicity of the dissipation representation. In addition to the first and second laws of thermodynamics, this framework imposes constraints from Onsager–Casimir reciprocal relationships [51,52,53] to ensure time-reversal symmetry near equilibrium. In addition to providing a methodology to check for thermodynamic consistency, this method of derivation simultaneously reduces the number and nature of phenomenological transport parameters. This framework has been useful in generating continuum-level descriptions of numerous systems with disparate physics (e.g., see the monograph by Beris and Edwards [43] and the more recent reviews [44,45]).

Ever since its inception, the development of better models to describe mesoscale dynamics has always been a significant thrust of inquiry among the nonequilibrium thermodynamics community [43,44,45,46,48,49,50,54,55,56]. More recently, there has been an increase in efforts to describe the entropy changes involved during fluctuations, nucleation, growth and self-assembly at the mesoscale level [56,57,58,59]. Nonequilibrium thermodynamics has been proven to be a useful tool in identifying degrees of freedom required to model the mesoscale dynamics [60]. In the present work, we try to accomplish a more systematic connection between a well-defined microstructure and nonequilibrium thermodynamics for the particular case of a thixotropic aggregating particle suspension. To achieve this goal, we employ the SGBF-NET framework to unify a conformation-based viscoelastic tensorial model with a population-balance-based kinetics model, thereby providing rigorous coupling of the structural variables and the viscoelastic material properties. In doing so, we achieve a new theoretical framework combining viscoelastic modeling of thixotropy [61,62] with a population-based structural analysis [3,40], all within a nonequilibrium thermodynamic framework. In doing so, we assure the thermodynamic consistency of the resulting model. We further analyze the model predictions, providing evidence for the validity of the model in describing thixotropy and viscoelasticity in various rheological shear flow steady-state and transient tests.

The manuscript is organized as follows. In the next section (Section 2), the derivation of the model equations developed within the SGBF-NET framework is summarized. This is presented systematically, starting with the description of the state variables, the Hamiltonian functional and the Poisson and Dissipation brackets. As an illustration of the model, predictions for both steady and transient flows are provided and discussed in the context of the literature in Section 3. In Section 4, the corresponding expression for the entropy production is presented, which enables evaluating thermodynamic consistency. Finally, in Section 5, we present our conclusions.

## 2. Model Development

Within the SGBF-NET model presented by Beris and Edwards [43], there are four steps needed to develop the governing model equations. Those are: (1) the selection of the system state variables, $\underline{x}$, which are typically field quantities depending on space and time; (2) the description of the extended free energy, the Hamiltonian function, as a functional involving the system state variables, $\underline{x}$, $H\left[\underline{x}\right]$; (3) the formulation of the Poisson bracket, {⋅,⋅}, describing the reversible dynamics; (4) the formulation of the dissipation bracket, [⋅,⋅], describing the irreversible dynamics. Accordingly, one can describe the time evolution for any arbitrary functional $F\left[\underline{x}\right]$ as [43]:

(1) $$\frac{dF}{dt}=\left\{F,H\right\}+\left[F,H\right].$$

Application of the above relationships and a comparison with the expression obtained through differentiation of the following parts:

(2) $$\frac{dF}{dt}=\int_{\Omega }\frac{\delta F}{\delta \underline{x}}\cdot \frac{\partial \underline{x}}{\partial t}dV,$$

leads to the governing equations for the state variables (as in [43]).

### 2.1. State Variables

Macroscopic fluid properties, such as viscosity, elastic moduli and relaxation times, depend on the agglomerate size distribution. Under deformation, the evolution of the agglomerate size distribution results in complex rheological signatures, such as shear thinning, thixotropy and viscoelasticity. Capturing these dynamics requires an appropriate choice of state variables.

It is critically important to select state variables that compactly but accurately represent this size distribution, because the aggregating suspensions generally consist of a distribution of time- or rate-dependent agglomerate sizes. Other than directly tracking the size distribution function, one common method to obtain a more compact, closed-form description for continuous probability density functions is by using a finite number of moments:

(3) $$M_{k}=\int_{0}^{\infty }v^{k}\frac{n(v,t)}{n_{p}}\;dv,$$

where v is the number of primary particles involved in the agglomerate, n(v,t) is the particle number distribution density and $n_{p}$ is the (constant here) number density of primary particles that generate the agglomerates (analogous to monomers in a polymeric system). Based on this definition, the zeroth moment is simply the fraction of all the particles (agglomerates and primary) over the total number of primary particles and is primarily connected with the method of normalization of the particle number density. The size distribution of fractal agglomerates that form in suspensions with sufficiently high numbers of primary particles is self-preserving [63,64]. The more moments are added to the solution space, the more accurate the description of the size distribution will be. Alternatively, if one uses a known particle size distribution, using a few moments might be enough to determine all of the others. Therefore, the aggregate sizes can be defined as $\underline{x}=\left\{M_{0},M_{1},\cdots ,M_{\chi -1}\right\}$, where the (finite) number of moments χ is chosen such that the dynamics and size distribution of the colloidal suspension are adequately characterized.

A common challenge in using a moment distribution is the need for a closure approximation to enable the calculation of any arbitrary properties of the distribution, including moments not explicitly represented (see [65] for a discussion on moment methods and closures). In the following, we use a lognormal closure, although the method developed here can be used with any other closure rule. The lognormal distribution [66] is especially well suited to describe the particle size distribution [3] and has found successful applications in describing size distributions in particulate systems [67,68]. Mathematically, the lognormal distribution arises when a process is a series of independent accumulated changes or events. In the context of a particle population, these independent events can be thought of as a series of aggregation steps required to obtain a particle of a certain size. Because each of these events is considered independent, their probabilities are multiplicative and additive on a logarithmic scale. Application of the central limit theorem on the logarithm of these probabilities results in the lognormal distribution. This result is also supported by the solution of the population balance equation following the aggregation kernels for Brownian motion and shear flow derived by Smoluchowski [69].

For a random variable distributed on the (0,∞) support and constrained by a mean and standard deviation, the lognormal distribution is the maximum entropy distribution and can be fully defined using the first three moments [70]. To rigorously satisfy mass (or the volume of primary particles) conservation requirements, the distribution can be normalized by the first moment, $M_{1}$ by setting its value to 1. Under these considerations, it is possible to keep in the system-state vector just the zeroth and second moments that can in turn be used to parameterize the lognormal distribution:

(4) $$n(v)=\frac{M_{0}n_{p}}{v\sigma \sqrt{2\pi }}\exp\left(-\frac{{\left(\ln v-\ln v_{0}\right)}^{2}}{2\sigma^{2}}\right),$$

where the normalized mean size parameter $v_{0}$ is expressed as:

(5) $$v_{0}=\frac{1}{\sqrt{M_{0}{}^{3}M_{2}}},$$

and the variance parameter $\sigma^{2}$ is given as:

(6) $$\sigma^{2}=\ln\left(M_{2}M_{0}\right).$$

The remaining system variables are the overall fluid momentum density, $\underline{m}$, used to characterize the overall fluid motion, and a dimensionless conformation tensor density, $\underset{=}{c}$, used to characterize the elastic deformation of the network formed by the agglomerates. For simplicity, in this work we are assuming $\underset{=}{c}$ to be a dimensionless relative deformation of agglomerates with respect to the equilibrium configuration. As such, it still depends on the aggregate size distribution; moreover, the way it affects the energetics is through an elastic modulus that it is itself a strong function of the aggregate size distribution. It is through this coupling that thixotropy is primarily introduced in this model. Macroscopically, under slowly varying flow conditions, $\underset{=}{c}$ is related to the finger strain tensor $\underset{=}{B}=\underset{=}{E}\cdot {\underset{=}{E}}^{T}$, where $\underset{=}{E}$ is the deformation–strain tensor of the agglomerate network. Thus, for a simple, aggregating, homogeneous (i.e., constant $n_{p}$) and incompressible (i.e., constant ρ) colloidal system under isothermal flows, the state vector can be defined as $\underline{x}\equiv \left\{\underline{m},M_{0},M_{2},\underset{=}{c}\right\}$.

### 2.2. Hamiltonian

The single-generator description of nonequilibrium thermodynamics uses the Hamiltonian functional, $H\left(\underline{x}\right)$, written as a functional of the system state variables, $\underline{x}$. Recently, Stephanou et al. [42] used the single-generator formalism to model the dynamics of macromolecular and colloidal solutions. In line with their approach, one can express the extra (in addition to the equilibrium contributions) free energy component of the Hamiltonian function using the kinetics, $K_{en}(\underline{x})$, and the nonequilibrium Helmholtz free energy, $A(\underline{x})$, of the system as follows:

(7) $$H=K_{en}(\underline{x})+A(\underline{x}),$$

where the kinetic energy is given by:

(8) $$K_{en}(\underline{x})=\int_{\Omega }\frac{\underline{m}\cdot \underline{m}}{2\rho }\;dV.$$

The Helmholtz free energy on the other hand is slightly more complicated, as it has contributions from both the elastic Helmholtz free energy (entropy-driven) stored in the agglomerate network and the Helmholtz free energy of mixing (mixing-entropy-driven) involved in the formation of flocs or agglomerates, shown as follows:

(9) $$A(\underline{x})=\int a(\underline{x})\;dV=\int \left[a_{\mathrm{el}}(\underline{x})+a_{\mathrm{mix}}(\underline{x})\right]\;dV,$$

where $a_{\mathrm{el}}(\underline{x}),\;a_{\mathrm{mix}}(\underline{x})$ are the elastic and mixing Helmholtz free energy densities, respectively. In turn, the elastic Helmholtz free energy density can be expressed in terms of the dimensionless conformation tensor, $\underset{=}{c}$, following the upper-convected Maxwell (UCM) model [43,44,45] as:

(10) $$a_{el}(\underline{x})=\frac{G(\phi_{a})}{2}\left[\mathrm{tr}\left(\underset{=}{c}-\underset{=}{I}\right)-\ln\det\underset{=}{c}\right],$$

where $G(\phi_{a})$ is the modulus of elasticity arising from agglomerates and $\phi_{a}$ is the relative agglomerate size/volume parameter that depends on moments of the distribution. The mixing Helmholtz free energy density is connected to the entropy of mixing as $a_{\mathrm{mix}}(\underline{x})=-T\Delta s_{\mathrm{mix}}$. For a population of flocs, the mixing entropy is defined as [55]:

(11) $$\Delta s_{\mathrm{mix}}=-k_{B}n_{p}M_{0}\int_{0}^{\infty }\frac{n(v,t)}{n_{p}M_{0}}\ln\frac{n(v,t)}{n_{p}M_{0}}\;dv.$$

We can use the lognormal distribution function, as defined in Equation (4), to calculate the entropy of mixing as stated above from the equivalent form:

(12) $$\Delta s_{\mathrm{mix}\;}=-k_{B}n_{p}M_{0}E\left[\ln\frac{n(v,t)}{n_{p}M_{0}}\right]$$

where E[.] stands for the expectation value calculated based on the normalized form of the lognormal distribution for $\frac{n(v,t)}{n_{p}M_{0}}$. Upon simplification and expressing the result in terms of the moments of the lognormal distribution (see Appendix A for derivation) yields:

(13) $$\Delta s_{\mathrm{mix}}=k_{B}n_{p}M_{0}\left[\frac{1}{2}+\frac{1}{2}\ln\left(\frac{2\pi \ln M_{2}M_{0}}{M_{2}M_{0}}\right)-\ln M_{0}\right],$$

which can also be alternatively written in terms of the $\sigma^{2}$ parameter as follows:

(14) $$\Delta s_{\mathrm{mix}}=k_{B}n_{p}M_{0}\left[\frac{1}{2}+\frac{1}{2}\ln\left(\frac{2\pi \sigma^{2}}{\exp\sigma^{2}}\right)-\ln M_{0}\right].$$

Using the aforementioned expressions, the full Hamiltonian can now be constructed as:

(15) $$H(\underline{x})=\int_{\Omega }\frac{\underline{m}\cdot \underline{m}}{2\rho }\;dV+\int \left[\frac{G(\phi_{a})}{2}\left[\mathrm{tr}\left(\underset{=}{\tilde{c}}-\underset{=}{\delta }\right)-\ln\det\underset{=}{\tilde{c}}\right]-k_{B}Tn_{p}M_{0}\left[\frac{1}{2}+\frac{1}{2}\ln\left(\frac{2\pi \sigma^{2}}{\exp\sigma^{2}}\right)-\ln M_{0}\right]\right]\;dV$$

### 2.3. Poisson Bracket

Once the system variables are specified and their physical and tensorial characteristics have been identified, we can construct the Poisson bracket, as each system variable contributes according to its physical nature and tensorial character [43]. The Poisson bracket for this choice of system variables is defined for two arbitrary functionals, *F*, *G*, as:

(16) $$\begin{array}{cc} \left\{F,G\right\} & =-\int \left[\frac{\delta F}{\delta m_{\gamma }}\nabla_{\beta }\left(m_{\gamma }\frac{\delta G}{\delta m_{\beta }}\right)-\frac{\delta G}{\delta m_{\gamma }}\nabla_{\beta }\left(m_{\gamma }\frac{\delta F}{\delta m_{\beta }}\right)\right]dV-\int \left[\frac{\delta F}{\delta c_{\alpha \beta }}\nabla_{\gamma }\left(c_{\alpha \beta }\frac{\delta G}{\delta m_{\beta }}\right)-\frac{\delta G}{\delta c_{\alpha \beta }}\nabla_{\gamma }\left(c_{\alpha \beta }\frac{\delta F}{\delta m_{\beta }}\right)\right]dV\\ & +\int c_{\gamma \alpha }\left[\frac{\delta F}{\delta c_{\alpha \beta }}\nabla_{\gamma }\left(\frac{\delta G}{\delta m_{\beta }}\right)-\frac{\delta G}{\delta c_{\alpha \beta }}\nabla_{\gamma }\left(\frac{\delta F}{\delta m_{\beta }}\right)\right]dV+\int c_{\gamma \beta }\left[\frac{\delta F}{\delta c_{\alpha \beta }}\nabla_{\beta }\left(\frac{\delta G}{\delta m_{\alpha }}\right)-\frac{\delta G}{\delta c_{\alpha \beta }}\nabla_{\beta }\left(\frac{\delta F}{\delta m_{\alpha }}\right)\right]dV\\ & -\sum_{i=0,2}^{}\int \left[\frac{\delta F}{\delta M_{i}}\nabla_{\beta }\left(M_{i}\frac{\delta G}{\delta m_{\beta }}\right)-\frac{\delta G}{\delta M_{i}}\nabla_{\beta }\left(M_{i}\frac{\delta F}{\delta m_{\beta }}\right)\right]\;dV. \end{array}$$

Note that for convenience, here and in the following, we use Einstein’s repeated indices summation convention, i.e., whenever repeated Greek letter subscripts appear, those also imply a summation from 1 to 3. The Poisson bracket is an antisymmetric bilinear functional with respect to the functionals *F*, *G*, involving Volterra derivatives of those with respect to the state variable—see Appendix B for a brief definition and evaluation. It also satisfies the Jacobi identity [43]. As defined above, it involves contributions needed to specify the reversible dynamics for the momentum density (first integral on the right hand side), the upper-convected derivative of a contravariant second order tensor, the conformation tensor $\underset{=}{c}$ (next three terms) and the material derivatives of three scalars, as well as two moments ($M_{0},M_{2}$) of the size distribution of the aggregate particles (last term).

### 2.4. Dissipation Bracket

The primitive part of the dissipation bracket (i.e., without the entropy correction) is defined here as a symmetric bilinear form of functionals *F*, *G* in terms of the variables involved. Keeping the lowest order terms while also respecting the symmetries involved and the physical meaning of the variables involved (i.e., whether they are equilibrium or nonequilibrium variables) gives:

(17) $$\begin{array}{cc} {\left[F,G\right]}_{p} & =-\int \frac{\delta F}{\delta c_{\alpha \beta }}\Lambda_{\alpha \beta \gamma \varepsilon }^{\left(c\right)}\frac{\delta G}{\delta c_{\gamma \varepsilon }}\;dV-\int \nabla_{\alpha }\left(\frac{\delta F}{\delta m_{\beta }}\right)Q_{\alpha \beta \gamma \varepsilon }\nabla_{\gamma }\left(\frac{\delta G}{\delta m_{\varepsilon }}\right)\;dV\\ & -\sum_{i=0,2}\int \frac{\delta F}{\delta M_{i}}\Lambda_{M_{i}}^{\left(a\right)}\frac{\delta G}{\delta M_{i}}\;dV-\sum_{i=0,2}\int \Lambda_{M_{i}\alpha \beta }^{\left(b\right)}\left(\frac{\delta F}{\delta c_{\alpha \beta }}\frac{\delta G}{\delta M_{i}}+\frac{\delta F}{\delta M_{i}}\frac{\delta G}{\delta c_{\alpha \beta }}\right)\;dV. \end{array}$$

In the above expression, the fourth-order transport properties of the viscosity matrix, $Q_{\alpha \beta \gamma \varepsilon }$, and phenomenological parameters, $\Lambda_{\alpha \beta \gamma \varepsilon }^{\left(c\right)},\;\Lambda_{M_{i}}^{\left(a\right)},\;\Lambda_{M_{i}\alpha \beta }^{\left(b\right)}$, are introduced and their relations to the system variables become apparent. By requiring the equivalence of Equation (1) to Equation (2) for arbitrary functionals *F* and using the definitions provided in Equations (16) and (17), the governing equations for the evolution in time of the system variables can now be derived. The dissipation bracket contains the symmetric contributions signifying the coupled dynamics of various system-state variables. There can potentially be additional terms arising from the coupling between $M_{0}$ and $M_{2}$, but for simplicity of analysis, they have been left out.

### 2.5. Governing Equations

Following the four steps outlined above, the governing equations for the time evolution of the state variables are determined by requiring the equivalence of Equations (1) and (2) for an arbitrary functional F. Note that the pressure, P, is introduced as a Lagrange multiplier to the momentum equation as a result of enforcing the divergence-free constraint of the momentum density (and thereby also the Volterra derivatives with respect to the momentum density) due to the incompressibility assumption—see [43] for further details.

First, the momentum equation is obtained using both elastic and viscous stress contributions to the extra stress $\underset{=}{\sigma }$ as:

(18) $$\rho \frac{Dv_{\alpha }}{\partial t}=\underset{\nabla_{\beta }\;\sigma_{\alpha \beta }}{\underset{︸}{\nabla_{\beta }\left(2c_{\beta \gamma }\frac{\delta H}{\delta c_{\alpha \gamma }}\right)+\nabla_{\beta }\left(Q_{\alpha \beta \gamma \varepsilon }\nabla_{\gamma }\frac{\delta H}{\delta m_{\varepsilon }}\right)}}-\nabla_{\alpha }P.$$

Similarly, the evolution of conformation tensor is given as follows:

(19) $$\frac{\partial c_{\alpha \beta }}{\partial t}+v_{\gamma }\nabla_{\gamma }\;c_{\alpha }{}_{\beta }-c_{\gamma \alpha }\nabla_{\gamma }v_{\beta }-c_{\gamma \beta }\nabla_{\gamma }v_{\alpha }=-\Lambda_{\alpha \beta \gamma \varepsilon }^{\left(c\right)}\frac{\delta H}{\delta c_{\gamma }{}_{\varepsilon }}-\sum_{i=0,2}\Lambda_{M_{i}\alpha \beta }^{\left(b\right)}\frac{\delta H}{\delta M_{i}},$$

or more simply:

(20) $${\overset{\nabla }{c}}_{\alpha \beta }=-\Lambda_{\alpha \beta \gamma \varepsilon }^{\left(c\right)}\frac{\delta H}{\delta c_{\gamma }{}_{\varepsilon }}-\sum_{i=0,2}\Lambda_{M_{i}\alpha \beta }^{\left(b\right)}\frac{\delta H}{\delta M_{i}}.$$

where the overscript $\overset{\nabla }{○}$ on the left-hand side of Equation (20) represents the upper-convected derivative:

(21) $${\overset{\nabla }{c}}_{\alpha \beta }\equiv \frac{\partial c_{\alpha \beta }}{\partial t}+v_{\gamma }\nabla_{\gamma }\;c_{\alpha }{}_{\beta }-c_{\gamma \alpha }\nabla_{\gamma }v_{\beta }-c_{\gamma \beta }\nabla_{\gamma }v_{\alpha }.$$

Note that from our selection of the Poisson bracket, Equation (16), the upper-convected derivative naturally emerges. This corresponds to the most natural description of polymeric viscoelasticity [71] and is the one most appropriate for the physical interpretation of the conformation tensor as a relative deformation. Finally, the evolution equations for the microstructural moments ($M_{i},i=0,2$) are obtained as:

(22) $$\frac{DM_{i}}{dt}=-\Lambda_{M_{i}}^{\left(a\right)}\frac{\delta H}{\delta M_{i}}+\Lambda_{M_{i}\alpha \beta }^{\left(b\right)}\frac{\delta H}{\delta c_{\alpha \beta }};\;i=0,2.$$

The Volterra functional derivatives that appear in the above expressions are summarized in Appendix B. More specifically, when substituted in the above equation, Equation (22), the two equations describing the two moments of the size distribution become:

(23) $$\begin{array}{cc} \frac{DM_{0}}{dt} & =-\Lambda_{M_{0}}^{\left(a\right)}\left[-\frac{1}{2}n_{p}k_{B}T\left(-2-2\ln M_{0}+\ln\left(\frac{2\pi \sigma^{2}}{\exp\sigma^{2}}\right)+\frac{1}{\sigma^{2}}\right)+\frac{\partial G(\phi_{a})}{\partial M_{0}}\left(\mathrm{tr}\left(\underline{\underline{c}}-\underline{\underline{I}}\right)-\ln\det\underline{\underline{c}}\right)\right]\\ & \;+\frac{G(\phi_{a})}{2}{\underline{\underline{\Lambda }}}_{M_{0}}^{\left(b\right)}\cdot \left(\underset{=}{I}-{\underset{=}{c}}^{-1}\right), \end{array}$$

(24) $$\frac{DM_{2}}{dt}=-\Lambda_{M_{2}}^{\left(a\right)}\left[-\frac{1}{2}n_{p}k_{B}T\frac{M_{0}\left(1-\sigma^{2}\right)}{M_{2}\sigma^{2}}+\frac{\partial G(\phi_{a})}{\partial M_{2}}\left(\mathrm{tr}\left(\underset{=}{c}-\underset{=}{I}\right)-\ln\det\underset{=}{c}\right)\right]+\frac{G(\phi_{a})}{2}{\underset{=}{\Lambda }}_{M_{2}}^{\left(b\right)}\cdot \left(\underset{=}{I}-{\underset{=}{c}}^{-1}\right).$$

Using entropy maximization, the static (no flow) equilibrium values for the zeroths and second moment of the distribution can be evaluated as:

(25) $$\begin{array}{l} M_{0}^{eq}=\frac{\sqrt{2\pi }}{e},\\ M_{2}^{eq}=\frac{e^{2}}{\sqrt{2\pi }}. \end{array}$$

It is useful now to construct a dimensionless parameter, $\phi_{a}$, to characterize the volume of agglomerates, defined as the $3/d_{f}$ moment of the distribution ($d_{f}$ being the fractal dimension), which can be obtained easily from the lognormal closure:

(26) $$M_{k}=M_{0}v_{0}^{k}\exp\left(\frac{k^{2}\sigma^{2}}{2}\right)\Rightarrow \phi_{a}=M_{0}v_{0}^{3/d_{f}}\exp\left(\frac{9\sigma^{2}}{2d_{f}^{2}}\right).$$

At equilibrium, $\phi_{a}$ is purely a function of the fractal dimension:

(27) $$\phi_{a}^{eq}={\left(2\pi \right)}^{\frac{1}{2}-\frac{3}{2d_{f}}}\exp\left(\frac{9}{2d_{f}^{2}}+\frac{3}{2d_{f}}-1\right),$$

which reduces to $\phi_{a}^{eq}=1$ when the fractal dimension $d_{f}=3$. Although this dimensionless volume parameter follows similar scaling as the agglomerate volume fraction, it is important to note that this model does not incorporate any excluded volume effects, such as arrested dynamics and jamming, whereby the volume fraction plays a more critical role and offers a better description of the microstructure.

### 2.6. Transport Coefficients

The transport coefficients appearing in this model offer quite a bit of flexibility regarding their functional form and dependence on other state variables. In our case, using the extended White–Metzner model [43,72] a description of the relaxation time, $\tau_{R}(\mathrm{tr}\underset{=}{c},\phi_{a})$ that depends on the trace of conformation tensor and agglomerate size, one can construct the fourth order tensor $\Lambda_{\alpha \beta \gamma \varepsilon }^{\left(c\right)}$ as:

(28) $$\Lambda_{\alpha \beta \gamma \varepsilon }^{\left(c\right)}=\frac{2}{\tau_{R}(\mathrm{tr}\underset{=}{c},\phi_{a})G(\phi_{a})}\left(c_{\alpha \gamma }\delta_{\beta \varepsilon }+c_{\alpha \varepsilon }\delta_{\beta \gamma }+c_{\beta \gamma }\delta_{\alpha \varepsilon }+c_{\beta \varepsilon }\delta_{\alpha \gamma }\right).$$

This choice represents the simplest one able to describe the Maxwell-based shear thinning viscoelasticity. More involved expressions are also available [43,44,45]. We chose that one for its simplicity; other more involved expressions can be used in the future, depending on the needs, to model specific data. The tensors that couple the moments of the distribution and the conformation tensor are assumed to have a simple linear dependence on the conformation tensor, leading to:

(29) $$\Lambda_{M_{0}\alpha \beta }^{\left(b\right)}=\frac{2}{n_{p}k_{B}T\tau_{M_{0}}^{\left(b\right)}}c_{\alpha \beta };\;\Lambda_{M_{2}\alpha \beta }^{\left(b\right)}=\frac{2M_{2}}{n_{p}k_{B}T\tau_{M_{2}}^{\left(b\right)}M_{0}}c_{\alpha \beta }.$$

In physical terms, the relaxation parameters $\Lambda_{M_{0}\alpha \beta }^{\left(b\right)},\Lambda_{M_{2}\alpha \beta }^{\left(b\right)}$ connect changes in the microstructure to viscoelastic time scales. It is to be noted that many other functional forms of the phenomenological constants are admissible, provided they do not violate properties of the dissipation bracket. This flexibility allows one to accommodate a more realistic microstructure dependence, while ensuring that the resulting model will be thermodynamically consistent. The relaxation parameters $\Lambda_{M_{i}}^{\left(a\right)}$ can be defined as:

(30) $$\Lambda_{M_{0}}^{\left(a\right)}=\frac{2}{n_{p}k_{B}T\tau_{M_{0}}^{\left(a\right)}}M_{0}^{2};\;\Lambda_{M_{2}}^{\left(a\right)}=\frac{2M_{2}}{n_{p}k_{B}T\tau_{M_{2}}^{\left(a\right)}M_{0}}M_{2}.$$

Finally, the fourth-order viscous dissipation tensor is assumed to correspond to that of an isotropic Newtonian viscous fluid [43] with a dependence on the agglomerate size:

(31) $$Q_{\alpha \beta \gamma \varepsilon }=\eta (\phi_{a})\left(\delta_{\alpha \gamma }\delta_{\beta \varepsilon }+\delta_{\alpha \varepsilon }\delta_{\beta \gamma }\right).$$

### 2.7. Final Form of Governing Equations

After substituting the above expressions for the transport coefficients, we can obtain the final form of the governing equations as:

(32) $${\overset{\nabla }{c}}_{\alpha \beta }=-\left[\begin{array}{l} \frac{1}{\tau_{M_{0}}^{\left(b\right)}}\left(2+2\ln M_{0}-\ln\left(\frac{2\pi \sigma^{2}}{\exp\sigma^{2}}\right)-\frac{1}{\sigma^{2}}\right)+\frac{1}{\tau_{M_{2}}^{\left(b\right)}}\frac{\left(\sigma^{2}-1\right)}{\sigma^{2}}\\ +\frac{2}{n_{p}k_{B}T}\left(\frac{1}{\tau_{M_{0}}^{\left(b\right)}}\frac{\partial G(\phi_{a})}{\partial M_{0}}+\frac{M_{2}}{\tau_{M_{2}}^{\left(b\right)}M_{0}}\frac{\partial G(\phi_{a})}{\partial M_{2}}\right)\left(\mathrm{tr}\left(\underset{=}{c}-\underset{=}{I}\right)-\ln\det\underset{=}{c}\right) \end{array}\right]c_{\alpha \beta }\;-\frac{1}{\tau_{R}(\mathrm{tr}\underline{\underline{c}},\phi_{a})}\left(c_{\alpha \beta }-\delta_{\alpha \beta }\right),$$

(33) $$\frac{DM_{0}}{dt}=-\frac{M_{0}^{2}}{\tau_{M_{0}}^{\left(a\right)}}\left[\begin{array}{l} 2+2\ln M_{0}-\ln\left(\frac{2\pi \sigma^{2}}{\exp\sigma^{2}}\right)-\frac{1}{\sigma^{2}}\\ +\frac{2}{n_{p}k_{B}T}\frac{\partial G(\phi_{a})}{\partial M_{0}}\left(\mathrm{tr}\left(\underset{=}{c}-\underset{=}{I}\right)-\ln\det\underset{=}{c}\right) \end{array}\right]+\frac{1}{\tau_{M_{0}}^{\left(b\right)}}\frac{G(\phi_{a})}{n_{p}k_{B}T}\mathrm{tr}\left(\underset{=}{c}-\underset{=}{I}\right),$$

(34) $$\frac{DM_{2}}{dt}=-\frac{M_{2}}{\tau_{M_{2}}^{\left(a\right)}}\left[\begin{array}{l} \frac{\left(\sigma^{2}-1\right)}{\sigma^{2}}\\ +\frac{2}{n_{p}k_{B}T}\frac{M_{2}}{M_{0}}\frac{\partial G(\phi_{a})}{\partial M_{2}}\left(\mathrm{tr}\left(\underset{=}{c}-\underset{=}{I}\right)-\ln\det\underset{=}{c}\right) \end{array}\right]+\frac{M_{2}}{\tau_{M_{2}}^{\left(b\right)}M_{0}}\frac{G(\phi_{a})}{n_{p}k_{B}T}\mathrm{tr}\left(\underline{\underline{c}}-\underline{\underline{I}}\right),$$

along with the following expression for the extra stress tensor $\underset{=}{\sigma }$:

(35) $$\underset{=}{\sigma }=G(\phi_{a})\left(\underset{=}{c}-\underset{=}{I}\right)+\eta (\phi_{a})\underset{=}{\dot{\gamma }}.$$

For the relaxation time $\tau_{R}(\mathrm{tr}\underset{=}{c},\phi_{a})$, we assume that it can be modeled through an extended White–Metzner power law with respect to the conformation tensor [43,72]:

(36) $$\tau_{R}(\mathrm{tr}\underset{=}{c},\phi_{a})=\tau_{R}^{eq}f\left(\phi_{a}\right){\left(\frac{\mathrm{tr}\underset{=}{c}}{3}\right)}^{-k}\equiv \frac{\tau_{R}^{eq}\tilde{\eta }(\phi_{a})}{\left(1-\frac{\phi_{a}}{\phi_{\max}}\right)}{\left(\frac{\mathrm{tr}\underset{=}{c}}{3}\right)}^{-k},$$

where $\tilde{\eta }(\phi_{a})=\eta (\phi_{a})/\eta_{s}$, and the exponent k determines the degree of shear thinning. If k is positive, the relaxation time will increase with deformation as the agglomerates will not be able to form strong connections [72]. Physically, this is a result of agglomerates not being able to form stronger bonds in flow conditions. The equilibrium relaxation time, $\tau_{R}^{eq}=\eta_{S}/G_{0}$, is the ratio of the solvent viscosity to the equilibrium elastic modulus, $G_{0}$.

The evolution equation for the conformation tensor, Equation (32), describes the upper-convected derivative of the conformation tensor in terms of two primary contributions, with the first one (first bracket in Equation (32)) arising from the last (fourth) integral contribution to the dissipation bracket, Equation (17), coupling structural moments with the viscoelasticity, while the second one arises from the White–Metzner relaxation time weighting of the deviation from the equilibrium configuration, $\left(c_{\alpha \beta }-\delta_{\alpha \beta })/\tau_{R}(\mathrm{tr}\underset{=}{c},\phi_{a}\right)$. The ratio of weighting factors of $c_{\alpha \beta }$ determines which of these terms dominates at any instant in time. Around equilibrium, the dimensionless constants $\tau_{M_{0}}^{\left(b\right)}/\tau_{R}^{eq}$ and $\tau_{M_{2}}^{\left(b\right)}/\tau_{R}^{eq}$ can be used to estimate this ratio. For lower values of $\tau_{M_{0}}^{\left(b\right)}/\tau_{R}^{eq}$ and $\tau_{M_{2}}^{\left(b\right)}/\tau_{R}^{eq}$, the first term dominates, indicating that faster breakage diminishes strong viscoelastic effects that emerge from agglomerate networks, and the nonlinear effects become prominent resulting in a departure from linear viscoelasticity. Likewise, at higher values of $\tau_{M_{0}}^{\left(b\right)}/\tau_{R}^{eq}$ and $\tau_{M_{2}}^{\left(b\right)}/\tau_{R}^{eq}$, the network contribution to the viscoelastic effects becomes prominent and the fluid follows linear viscoelastic scaling. The dynamics of mesoscale structure evolution and viscoelasticity are governed by separate timescales, making this model distinct from nonlinear time-dependent viscoelastic models with structural parameters, such as the one proposed by Acierno et al. [73], which are not considered to be true thixotropy models [12].

The structural moment evolution equations, Equations (33) and (34), have similar forms in their right hand sides consisting of aggregation and breakage terms that depend on the moments as well as on the conformation tensor. The first term (involving a bracket) is weighted by a time constant $\tau_{M_{i}}^{\left(a\right)}$ and governs the aggregation. The first and second rows in the bracketed term in Equation (33) correspond to the contributions from Brownian aggregation and deformation-driven aggregation, respectively (same applies to Equation (34)). The last term in Equations (33) and (34) is weighted by the time constant $\tau_{M_{i}}^{\left(b\right)}$ and describes the breakup of agglomerates.

It is important to note here that in Equations (33) and (34), the breakage terms are scaled with $\mathrm{tr}\left(\underline{\underline{c}}-\underline{\underline{I}}\right)$ instead of the shear rate $\dot{\gamma }$ as proposed in other studies [12,16,22]. It has been shown that breakage terms that scale with $\dot{\gamma }$ also admit thixo-elastic materials in their framework. Larson [12] has shown thixo-elastic materials to be in violation of the second law of thermodynamics. In contrast, the breakage rate in this model is related to the elastic stress developed in the fluid. This idea is consistent with the mechanistic understanding of the breakage process (see the recent work by Joshi [19]). The elastic energy available to the agglomerates from the developed internal stress increases the probability of rearrangements and breakage. The primary particles and small clusters have enough available energy to break out of the interparticle potential’s attraction.

The governing equations involve nonlinear dependences that may prove difficult to resolve for all parameter values. Therefore, to simplify our analysis, we chose simple relationships for the elastic modulus and viscosity functions. The elastic modulus is established in the literature to be related to the concentration of primary particles in the agglomerates, and for a fractal structure, numerous scaling laws have been proposed in the literature, depending on the nature of interactions between the flocs. More commonly, the scaling law proposed by Shih et al. [5] is used. Marangoni [7] proposed a similar scaling law derived from a thermodynamic approach by relating the elasticity to the free energy changes arising from floc–floc interactions. The elastic modulus was found to scale as:

(37) $$G\left(\phi_{a}\right)=G_{0}\phi_{a}^{\frac{3}{3-d_{f}}}$$

for an agglomerate that forms three-dimensional networks. For the viscosity, the Maron–Pierce equation [74]:

(38) $$\eta \left(\phi_{a}\right)=\eta_{s}{\left(1-\frac{\phi_{a}}{\phi_{\max}}\right)}^{-2},$$

was used. More complex relations for viscosity have been developed over the years for dense suspensions with polydisperse particles, such as in the study by Mwasame et al. [75]. However, for simplicity of illustration, this common form with a constant maximum agglomerate volume is assumed.

The primary emphasis of this work is (a) establishing the theoretical foundation in NET for modeling a distribution of agglomerate sizes and (b) providing a route to independently incorporate developed models in a thermodynamically consistent manner. For a homogeneous system, the model has a total of 10 parameters: five time constants, $\tau_{M_{0}}^{\left(a\right)},\tau_{M_{2}}^{\left(a\right)},\tau_{M_{0}}^{\left(b\right)},\tau_{M_{2}}^{\left(b\right)},\tau_{R}^{eq}$; two elasticity moduli, $n_{p}k_{B}T,\;G_{0}$; and three dimensionless numbers, $\phi_{\max},\;d_{f},\;k$. For simplicity, we assume common aggregation and breakage times for the moments here $\tau_{M_{0}}^{\left(a\right)}=\tau_{M_{2}}^{\left(a\right)}\equiv \tau_{a}$, $\tau_{M_{0}}^{\left(b\right)}=\tau_{M_{2}}^{\left(b\right)}\equiv \tau_{b}$ and a typical scaling for the maximum strength of the elastic modulus $G_{0}=n_{p}k_{B}T$, which reduces the total to seven parameters. In the following, we offer examples of the model predictions for steady and transient shear and extensional flows as obtained with indicative sets of model parameters.

## 3. Model Predictions

The model parameters are selected to illustrate behavior that aligns with physically observable systems. For simplicity, we fix the fractal dimension to that commonly observed for reaction limited aggregation, $d_{f}=2.1$ [33]. We also assign a maximum value to the maximum agglomerate volume parameter, $\phi_{\max}=2.7$, to set a threshold where there is a significant increase in viscosity of the suspension as the agglomerate volume approaches this value. Note that $\phi_{a}$ can be greater than unity, as it is an effective volume swept out by the agglomerates, and as such represents a volume containing both fluid and particles. For instance, when $d_{f}=3$, the volume occupied by the agglomerates is compact and reaches the equilibrium value of $\phi_{a}=1$. On the other hand, when the fractal dimension is lower, $\phi_{a}$ can be larger, reflecting the effective volume swept out by agglomerates that have a comparatively open structure. The connection of this agglomerate volume to the actual physical volume of the particle phase (or any other measurable physical property of the particle phase) is readily achieved through the particle agglomerate distribution function itself, which is defined by the moments for any state of the system. As a further simplification for purposes of illustration, we use $G_{0}$ and $\tau_{R}^{eq}$ to scale the stress and time results, respectively. These choices leave just three parameters to vary: the exponent k (which is already dimensionless) and two characteristic times, which can be conveniently substituted by the dimensionless time constants, $\lambda_{ba}\equiv \tau_{b}/\tau_{a}$ and $\lambda_{Ra}\equiv \tau_{R}^{eq}/\tau_{a}$, to demonstrate the model behavior for homogeneous steady and transient flows. These simplifications allow the presentation of the results for a generic thixotropic aggregating colloidal system. In particular, no effort is made to optimize the transport functions and fit the material parameters to any particular experimentally determined real system behavior.

### 3.1. Steady Shear Flow

The governing equations, Equations (32)–(35), are solved by assuming homogenous flow under simple shear conditions, with flow kinematics given by $\underline{v}=\left\{\dot{\gamma }y,0,0\right\}$, where $\dot{\gamma }$ is the constant shear rate. This is nondimensionalized as $Wi=\tau_{R}^{eq}\dot{\gamma }$. Simple shear flow only requires the solution of four differential equations, namely ${\dot{c}}_{11},{\dot{c}}_{12},{\dot{M}}_{0},{\dot{M}}_{2}$, given by Equations (32)–(34), simplifying the analysis. The $c_{22}$ component of the conformation tensor remains at its equilibrium value of unity. The MATLAB ordinary differential equation solver *ode23s* is for the steady state following a shear start-up transient, simultaneously confirming the stability of the presented results.

**Figure 2:**
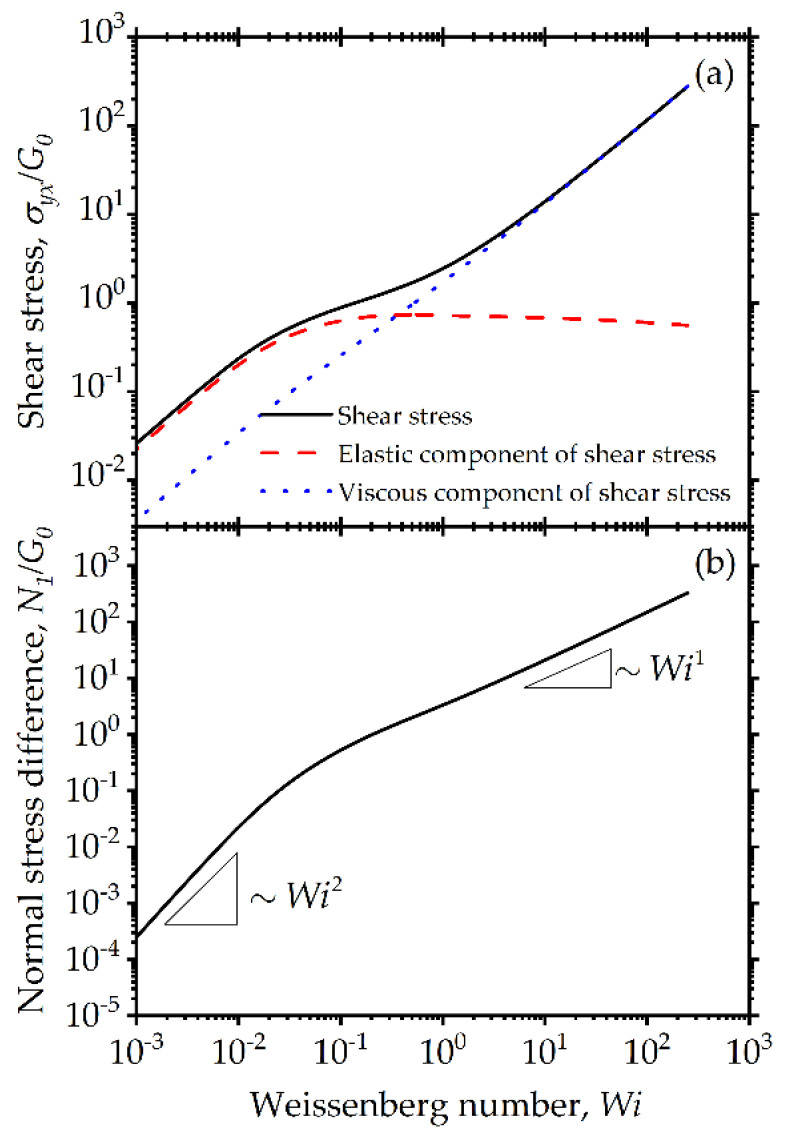
Model predictions for steady-state simple shear flow: (**a**) shear stress: total (solid line), elastic component (dashed line) and viscous component (dotted line); (**b**) first normal stress difference. The model parameters are: $\lambda_{ba}=100,\;\lambda_{Ra}=0.5,\;k=0$.

**Figure 3:**
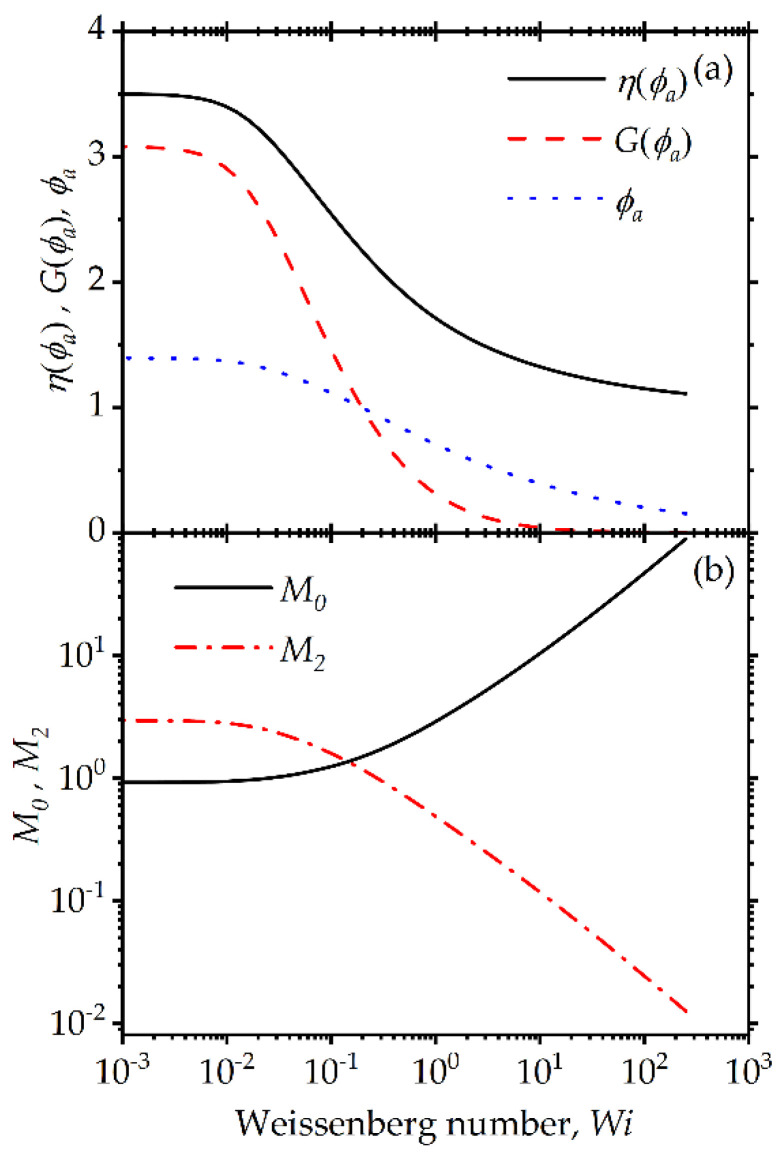
Model predictions for steady-state simple shear flow: (**a**) dimensionless viscosity (solid line), elastic modulus (dashed line) and agglomerate volume parameter (dotted line); (**b**) zeroth moment (solid line) and second moment (dot-dashed line) of the aggregate size distribution. The model parameters are: $\lambda_{ba}=100,\;\lambda_{Ra}=0.5,\;k=0$.

The shear stress and first normal stress difference are shown in Figure 2. As can be expected for viscoelastic materials, at very low Wi, Wi≤0.01, a purely Maxwellian viscoelastic behavior is observed, characterized by a linearly increasing stress and a quadratically increasing first normal stress difference [76]. However, we can also see an apparent yield stress if we only examine the limiting behavior at a small but finite shear rate [61,62]. Indeed, for Wi>0.01, we can see all characteristics of a typical viscoplastic colloidal suspension behavior. If the low shear viscoelastic regime is neglected, measurements for Wi>0.1 only would suggest the possibility of yield stress. If experimental information obtained at lower shear rate values is available suggesting the presence of a real yield stress, this can be introduced through a more complicated model, for example following the previous work by Beris et al. [77], although this is left for future work. At higher Wi values, the first normal stress difference grows linearly with the shear rate and remains positive. This is due to the dependence of the elastic modulus on the size distribution and the overall coupling of the size distribution with the conformation tensor. This nonlinear transition from Maxwellian to viscoplastic behavior is also evident in the viscosity plot shown in Figure 3a. As shown, around this critical value of Wi≈0.01 shear thinning of the viscosity is apparent with increasing Wi, followed by an even more dramatic reduction in elastic modulus, $G(\phi_{a})$ as the elasticity of the system is significantly disrupted by the breakage of agglomerates, which is also consistent with the significant reduction observed in $\phi_{a}$.

The changes in the agglomerate distribution with increasing steady shear rate are plotted in Figure 3b, presented as the behavior of the zeroth and second moments of the agglomerate size distribution with increasing Wi. The zeroth moment, $M_{0}$, represents the number of agglomerates per unit volume. As the structure breaks down, the number of agglomerates is expected to rise, as evident in the model predictions, and due to their fractal nature, as their average size decreases their effective volume decreases as well. The second moment of the distribution, which is related to the width of the distribution, is expected to decrease because at higher deformation rates, the agglomerate distribution asymptotically approaches monodispersity. In this highly simplified model illustration, the highest agglomerate polydispersity is evident at equilibrium, and this evolves toward a monodisperse suspension of primary clusters at high shear rates.

**Figure 4:**
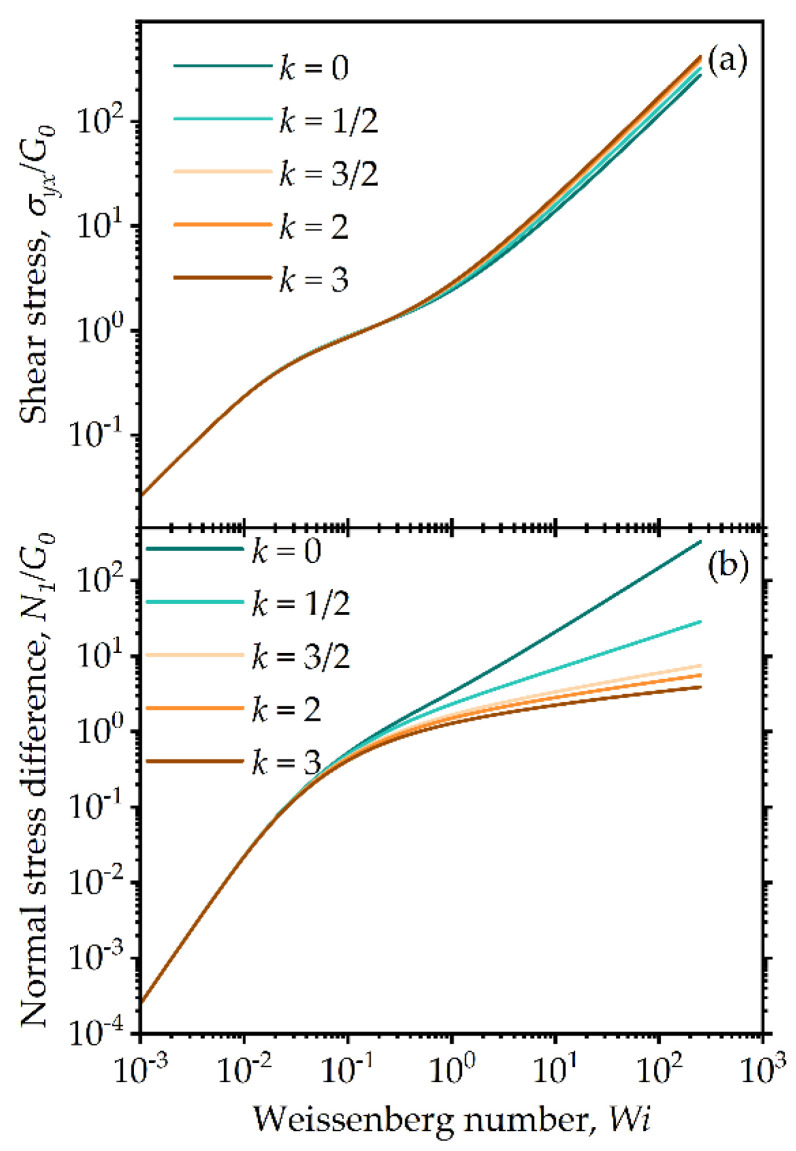
Model predictions for steady-state simple shear flow: (**a**) steady shear and (**b**) normal stress differences for different exponent values in Equation (36) (model parameters $\lambda_{ba}=100,\;\lambda_{Ra}=0.5$).

Of further interest is how the results change with a systematic change of one of the three main parameters of the model. Figure 4 is a plot of the shear stress and normal stress difference vs. Weissenberg numbers for various White–Metzner power law scaling values in Equation (36), shown as *k*. By changing the exponent k, we can affect the effective viscoelastic relaxation time. This has a small, systematic effect on the higher shear stress (Figure 4a), but a much more significant effect on the first normal stress difference, which reduces by several orders of magnitude, as shown in Figure 4b. The reason for the apparent insensitivity of the total shear stress is that this is dominated by the viscous contribution, whereas k controls the elastic contribution; inversely, the normal stress difference arises as a result of elastic effects. Such model predictions of parameter sensitivity can aid in parameter estimation when fitting to experimental data.

**Figure 5:**
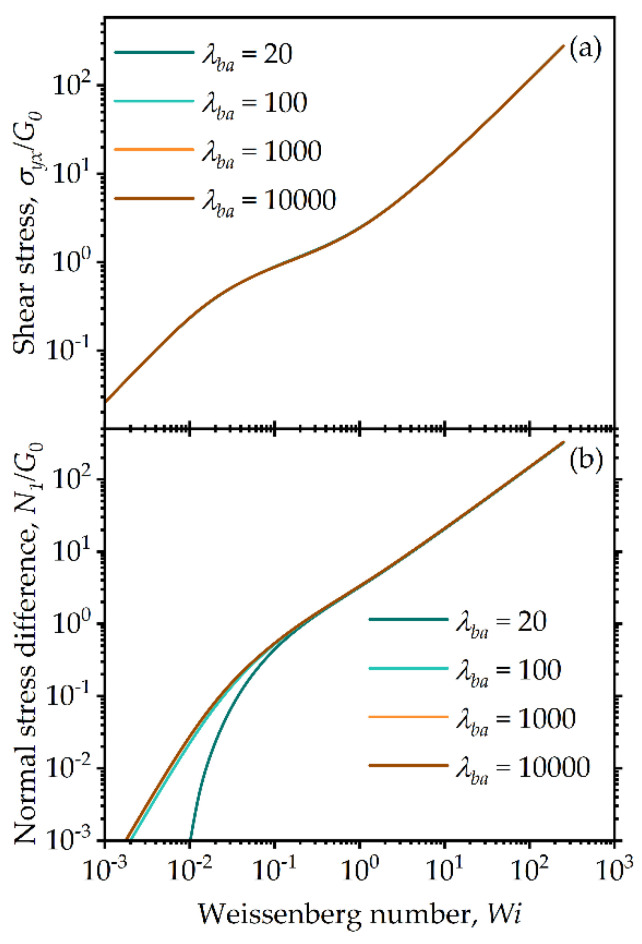
Model predictions for steady-state simple shear flow: (**a**) shear stress and (**b**) first normal stress difference for different values of the model parameter $\lambda_{ba}$ for $\lambda_{Ra}=0.5,\;k=0$.

**Figure 6:**
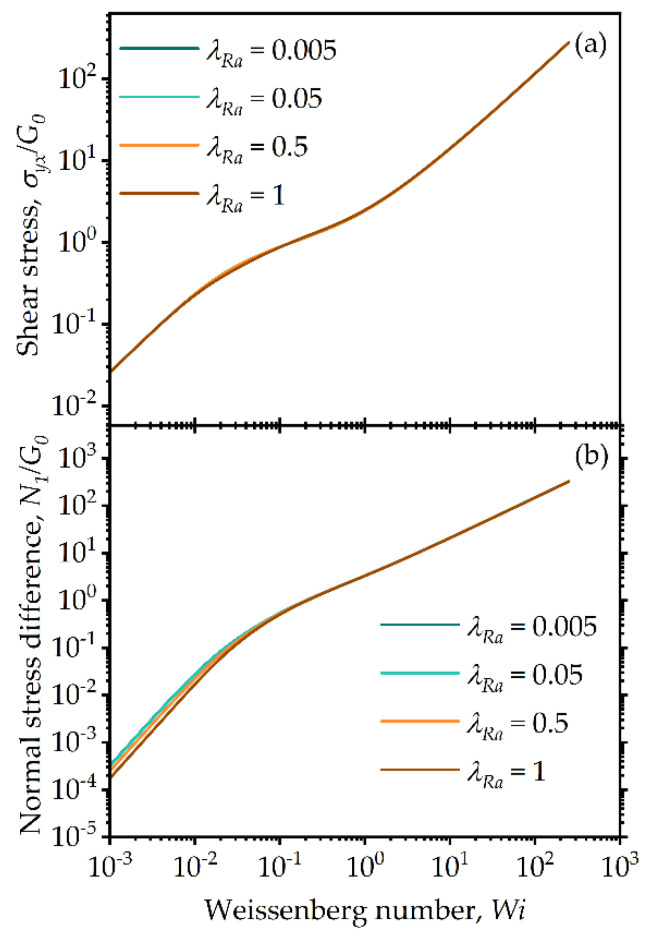
Model predictions for steady-state simple shear flow: (**a**) shear stress and (**b**) first normal stress difference for different values of the model parameter $\lambda_{Ra}$ for $\lambda_{ba}=100,\;k=0$.

Similar observations regarding changes in the model predictions with the model parameter $\lambda_{ba}$ are shown in Figure 5 and with $\lambda_{Ra}$ shown in Figure 6. As can be seen, the highest sensitivity is experienced again by the normal stress difference. The parameter ranges for $\lambda_{ba}$ and $\lambda_{Ra}$ are chosen to obtain reasonable thixotropic transient behavior, such that the difference between aggregation and breakage rates are within a few orders of magnitude for the range of Weissenberg numbers chosen, and they are limited from thermodynamic constraints, as explained in detail in Section 4. Of interest is the observation that a departure from the linear viscoelastic quadratic behavior for low Wi is observed for the lowest value used for the model parameter $\lambda_{ba}$ in Figure 5b. This is attributed to the fact that as this parameter decreases the nontraditional viscoelastic relaxation term becomes important (first term on the right hand side of Equation (32)).

### 3.2. Transient Shear Flow

**Figure 7:**
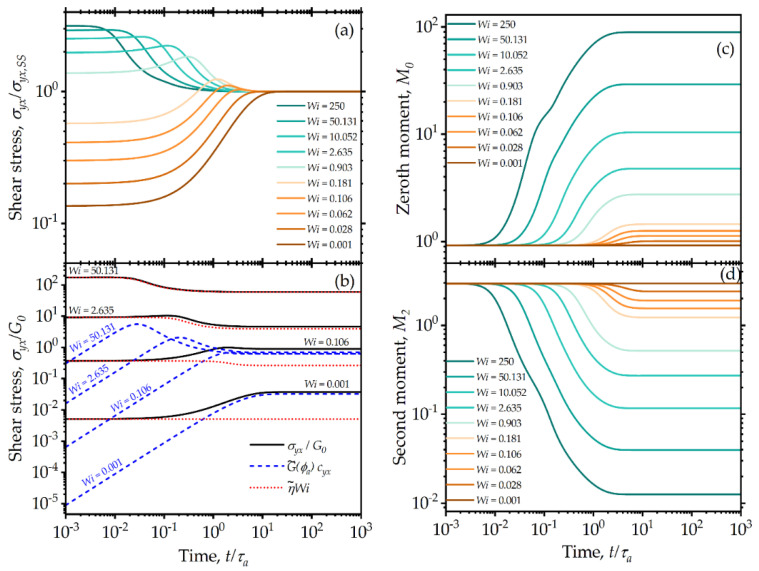
Model predictions for steady shear start-up transients from a quiescent condition subjected to different shear rates. The different curves correspond to increasing Wi values, increasing from the bottom to top curves, with values as indicated in the insert legend: (**a**) shear stresses, scaled by their final steady-state values; (**b**) total shear stress along with viscoelastic and viscous components for indicated Wi values; (**c**) zeroth and (**d**) second moments of the agglomerate size distribution. The model parameters are: $\lambda_{ba}=100,\;\lambda_{Ra}=0.5,\;k=0$.

Transient features of the model behavior help to distinguish thixotropy from pure shear thinning and time-dependent viscoelasticity. Therefore, transients are also important from a modeling standpoint. The present model predictions follow expected trends for typical transient experiments [11]. Plotted in Figure 7 are the shear stress (Figure 7a) and its elastic and viscous components (Figure 7b), along with the evolution of zeroth (Figure 7c) and second (Figure 7d) moments of the distribution when the fluid is subjected to a start-up of shear deformation from a quiescent equilibrium state ($M_{0}=M_{0}^{eq}=\sqrt{2\pi }/e;\;M_{2}=M_{2}^{eq}=e^{2}/\sqrt{2\pi };\;c_{11}=1;\;c_{22}=0$). In Figure 7a, the results are scaled with the corresponding steady-state values (Figure 2). For low Wi values, the stress growth follows that of viscoelastic fluid, i.e., an instantaneous buildup of stress due to viscous contribution, followed by a long transient as it takes a finite time for the fluid to relax its stress and for the breakdown in its structure to take place. The viscoelastic contribution to the stress (tracked through the conformation tensor) develops as the agglomerates deform with time under the applied deformation rate (Figure 7b). The evolution behavior is in line with that of the upper-convected Maxwell model but with nonlinear effects as a result of the more complex effective relaxation time due to the presence of multiple internal characteristic times affecting the time evolution of both the structure and deformation within the material in a complex nonlinear fashion. The model does not exhibit an explicit yielding behavior and always predicts small but significant viscous stress, even over short time periods. This is in agreement with experimentally observed yielding behavior in colloidal systems, where finite initial viscous responses result in sublinear initial stress growth [78]. The viscoelastic component of stress very closely follows the viscoelastic model with the structural parameters developed by Acierno et al. [73]. The structure breakdown is delayed for lower deformation rates because of the competing aggregation process that dominates the evolution of the agglomerate size (Figure 7c). The second moment of the distribution, which accounts for the variance in agglomerate sizes, reduces to a lower value for higher Wi, as shown in Figure 7d. The time required to attain a steady state is also seen to follow a similar trend, where the system attains a steady state much earlier at higher deformation rates. Physically, this occurs because agglomerates have greater mobility at higher deformation rates, allowing them to undergo rearrangement more quickly. A steady state is attained when the breakage and aggregation processes achieve dynamic equilibrium.

**Figure 8:**
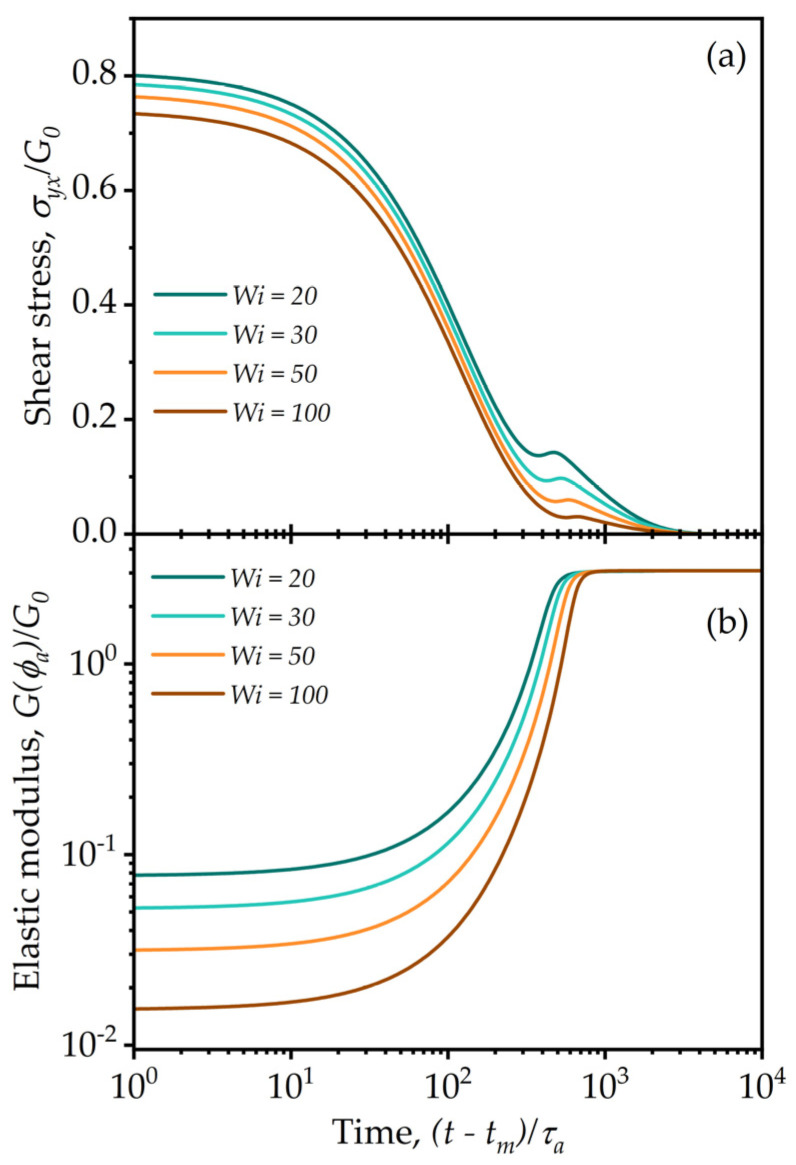
Model predictions of relaxation upon cessation of steady shear flow from an initial deformation rate indicated by Weissenberg number, with the curves corresponding to decreasing Wi values from bottom to top, with values as indicated in the insert legend. The fluid is initially subjected to steady shear from a quiescent state and allowed to attain steady state. Once that is attained, the deformation rate is set to zero. The evolution of the (**a**) shear stress and (**b**) elastic modulus after flow cessation is reported as a function of the time since the time $t=t_{m}$ when the flow is stopped. The model parameters are: $\lambda_{ba}=100,\;\lambda_{Ra}=0.5,\;k=0$.

The next investigation is the opposite of shear start-up, stress relaxation. As shown in Figure 8a, when the flow is stopped from steady state at fixed Wi the stress does not decay instantly because the fluid stores some elastic energy in the agglomerates and this requires finite time to relax (White–Metzner relaxation time), but as this relaxes the aggregation terms grow the agglomerate back toward the equilibrium distribution. Consequently, as the external deformation rate is set to zero and the stress relaxes, the material stiffens with time because of restructuring and aggregation that manifests as an increase in the elastic modulus, as shown in Figure 8b. The competition between the lowering of elastic deformation and increase in elastic modulus result occasionally in a nonmonotonic stress relaxation behavior, as seen in Figure 8a for *Wi* = 3. This phenomenon has been reported experimentally by Hendricks et al. [79] shown to be admissible thermodynamically under structure kinetic formalism by Joshi [19]. It is to be noted that Hendricks et al. study these effects in a polymeric solution where the effects of restructuring are much stronger when compared to a particulate suspension (as seen in Figure 8a where the overshoot is significantly smaller in magnitude compared to the initial value of stress).

**Figure 9:**
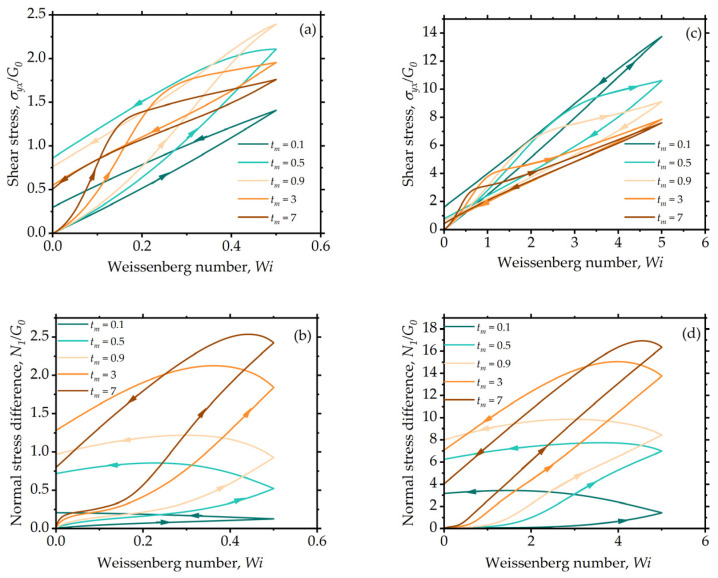
Model predictions for triangular shear transients. Hysteresis loops for shear stress (**a**,**c**) and first normal stress difference (**b**,**d**) for different values of $t_{m}$ plotted for $Wi_{\max}=0.5$ in (**a**,**b**) and $Wi_{\max}=5$ in (**b**,**d**). The model parameters are: $\lambda_{ba}=5,\;\lambda_{Ra}=0.5,\;k=0$.

Another transient feature that distinguishes thixotropy is the hysteresis observed in shear stress when the fluid is subjected to a ramp-up in deformation rate, followed by a ramp down at the same rate, such that:

(39) $$Wi(t)=\{\begin{array}{cc} Wi_{\max}\frac{t}{t_{m}} & 0\leq t<t_{m}\\ Wi_{\max}\left(2-\frac{t}{t_{m}}\right) & t_{m}\leq t\leq 2t_{m} \end{array},$$

where $t_{m}$ is the time when the deformation rate and Wi are at their maximum values. This procedure, also known as the triangular ramp test, results in the characteristic hysteresis loops, as seen in Figure 9. The loops for shear stress, shown in Figure 9a,c, as well as those for the first normal stress difference, shown in Figure 9b,d, are distinctly asymmetric, as a result of the changes taking place in the underlying structure that imparts elastic–viscoplastic–thixotropic behavior to the system. In Figure 9a, the Weissenberg number predominantly lies in the viscoelastic regime where the fluid retains a significant amount of stress even after the ramp cycle is completed. In the case of higher $Wi_{\max}$, the shear stress is higher during the ramp-up compared to ramp down because of the breakage of agglomerates, as shown in Figure 9c. Importantly, ramp conditions can generate curves with crossing of the stress, which are often observed in real systems under conditions where the kinetics of the structure breakdown and build-up are comparable to the ramp rate. Qualitatively, the results indicate that the model can capture a variety of transient behaviors that correspond to the hysteresis loops reported in the literature [10,12,22].

**Figure 10:**
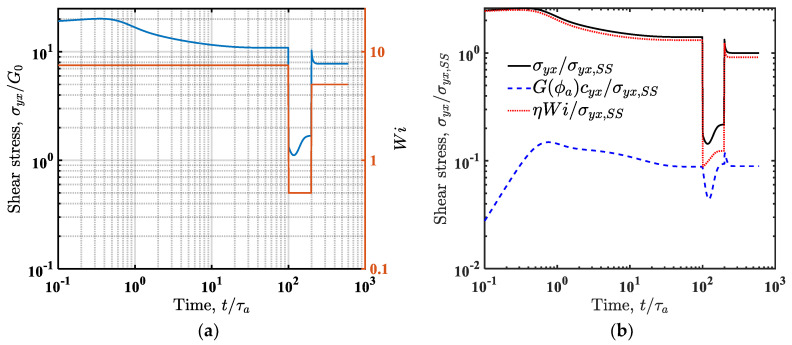
(**a**) Stress response of the model for parameters $\lambda_{ba}=100,\;\lambda_{Ra}=5,\;k=0$ for an intermittent shear rate step test, where a shear rate corresponding to Wi=7.5 is applied on a fluid at equilibrium, followed by a step down in shear rate to Wi=0.5 at $t/\tau_{a}=100$, and finally a step up in shear rate to Wi=5 at $t/\tau_{a}=200$. The applied deformation rate is depicted by the dotted orange line. The component-wise contribution to the total shear stress (scaled by its steady-state value) is shown in (**b**). The dashed blue line depicts the contribution from the viscoelastic term and the dotted red line is the viscous contribution, showing the inelastic thixotropy independent of the viscoelastic term.

The simplest evidence we can provide for thixotropy in the model is by performing an intermittent shear rate step test, where a shear rate is applied as depicted (as *Wi*) with an orange line in Figure 10a. From a high value of 7.5, it decreases to a low value of 0.5 at a dimensionless time of 100 before increasing to an intermediate value of 5 at a dimensionless time of 200. In addition to following the total stress over time, as shown in Figure 10a, where one can see a doubly nonmonotonic behavior, it is also interesting to follow its elastic and viscous contributions, shown in Figure 10b, which clearly explain the local minimum in the intermediate time range of 100–200 as being due to the elastic contribution arising from the evolving time microstructure, a concrete thixotropic phenomenon.

### 3.3. Uniaxial Elongational Deformation

**Figure 11:**
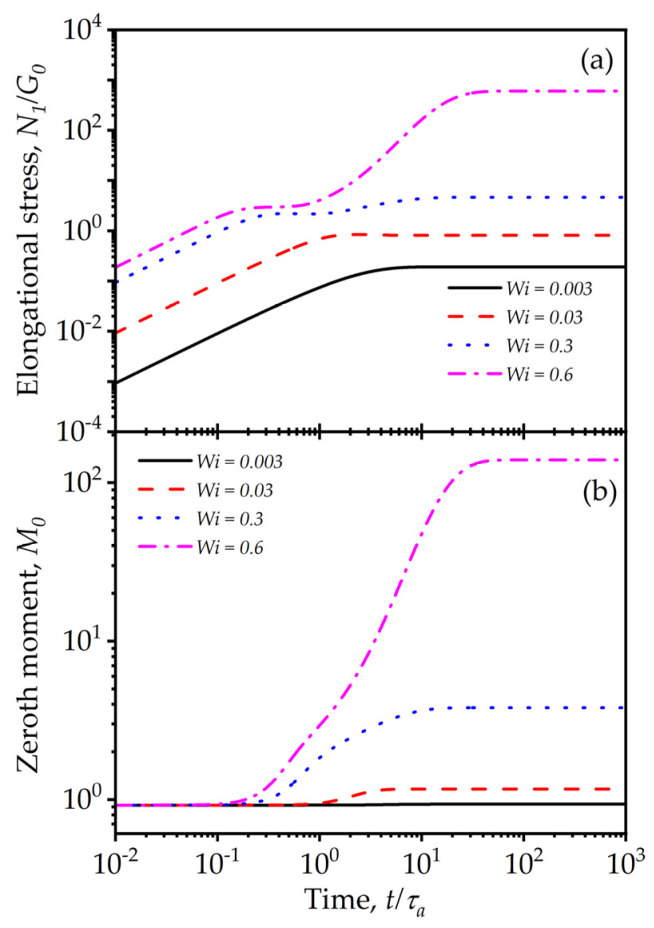
Model predictions for start-up transients for uniaxial elongation for different Weissenberg numbers, $Wi_{\varepsilon }=\tau_{R}^{eq}\dot{\varepsilon }$: (**a**) first normal stress difference; (**b**) zeroth moment. The model parameters are: $\lambda_{ba}=2,\;\lambda_{Ra}=0.3,\;k=0$.

The uniaxial elongation corresponds (in an *x, y, z* Cartesian coordinate system) to the flow, such that $\underline{v}=\left\{\dot{\varepsilon }x,-\frac{1}{2}\dot{\varepsilon }y,-\frac{1}{2}\dot{\varepsilon }z\right\}$, where $\dot{\varepsilon }$ is the rate of elongation. To evaluate the transients in uniaxial elongation, Equations (32)–(34) are solved for the diagonal components of the conformation tensor $c_{11},c_{22},c_{33}$ and the moments of the distribution, $M_{0},M_{2}$. Due to symmetry, $c_{22}$ and $c_{33}$ are identical. Figure 11a shows the first normal stress responses in a start-up of uniaxial elongation from a quiescent equilibrium state for different values of *Wi,* which is defined here as $Wi=\tau_{R}^{eq}\dot{\varepsilon }$. The stress growth is linear initially, indicating strong elastic behavior, approaching a stationary state followed by a yielding behavior, characterized by superlinear initial growth before subsiding to reach a steady state. The second yielding occurs as the structure changes, as is clearly evident in Figure 11b.

Note that a separate set of model parameters is used in the calculations reported in Figure 11 to show the most interesting model behavior. For most physical systems, the elongational yielding will be nearly instantaneous, as the material develops a much greater magnitude of stress in elongation compared to shear. Similar to the shear flow cases presented in Figure 4b, it is anticipated that changes to the exponent k may be critical in significantly shaping the stress predictions for elongational flow as well.

## 4. Entropy Generation and Thermodynamic Consistency

The SGBF-NET framework used to construct this model can also be used as an equation for the entropy production, which is an outcome of using the framework for the model derivation. Although the use of this framework itself does not thermodynamically guarantee consistency, it makes it convenient to determine the parameter ranges where the model makes thermodynamically consistent predictions, i.e., satisfies the criterion of non-negative entropy production. Because this is not always easy to evaluate analytically in a way that is valid for all occasions, it becomes important to perform numerical evaluations of selected flow cases (which of course provide a necessary but not sufficient condition for thermodynamic admissibility).

In the SGBF-NET model, the entropy correction ${\left[F,H\right]}_{ec}$ is related to the primary dissipation bracket ${\left[F,H\right]}_{p}\equiv \int_{\Omega }\Xi (F,H)\;dV$ in the following manner [43]:

(40) $${\left[F,H\right]}_{ec}=-\int_{\Omega }\frac{1}{\frac{\delta H}{\delta s}}\frac{\delta F}{\delta s}\Xi \left(H,H\right)\;dV,$$

with $\frac{\delta H}{\delta s}=T$. Using the procedure described in Appendix B, the total rate of entropy production can be obtained from the primitive dissipation bracket described in Equation (17) as:

(41) $$\sigma_{s}=\frac{1}{T}\left(\begin{array}{l} \overset{T\sigma_{s,v}}{\overset{︷}{\underline{\underline{\underline{\underline{Q}}}}::\underline{\nabla }\underline{v}\underline{\nabla }\underline{v}}}+\overset{T\sigma_{s,c}}{\overset{︷}{{\left(\frac{G\left(\phi_{a}\right)}{2}\right)}^{2}\left(\underline{\underline{I}}-{\underline{\underline{c}}}^{-1}\right):{\underline{\underline{\underline{\underline{\Lambda }}}}}^{\left(c\right)}:\left(\underline{\underline{I}}-{\underline{\underline{c}}}^{-1}\right)}}+\overset{T\sigma_{s,M_{0}}}{\overset{︷}{\Lambda_{M_{0}}^{\left(a\right)}{\left(\frac{\delta H}{\delta M_{0}}\right)}^{2}}}+\overset{T\sigma_{s,M_{2}}}{\overset{︷}{\Lambda_{M_{2}}^{\left(a\right)}{\left(\frac{\delta H}{\delta M_{2}}\right)}^{2}}}\\ +\underset{T\sigma_{s,cM_{0}}}{\underset{︸}{\frac{G\left(\phi_{a}\right)}{2}\left({\underline{\underline{\Lambda }}}_{M_{0}}^{\left(b\right)}:\left(\underline{\underline{I}}-{\underline{\underline{c}}}^{-1}\right)\frac{\delta H}{\delta M_{0}}\right)}}+\underset{T\sigma_{s,cM_{2}}}{\underset{︸}{\frac{G\left(\phi_{a}\right)}{2}\left({\underline{\underline{\Lambda }}}_{M_{2}}^{\left(b\right)}:\left(\underline{\underline{I}}-{\underline{\underline{c}}}^{-1}\right)\frac{\delta H}{\delta M_{2}}\right)}} \end{array}\right).$$

Calculation of the entropy production allows an illustration of the thermodynamic consistency of the proposed model. Following the notations shown in Equation (41), the first term, $\sigma_{s,v}$, representing the viscous entropy production, is always non-negative provided the viscosity is non-negative. The second term, $\sigma_{s,c}$, representing entropy production due to elastic relaxation, can be simplified to:

(42) $$T\sigma_{s,c}\equiv {\left(\frac{G\left(\phi_{a}\right)}{2}\right)}^{2}\left(\underline{\underline{I}}-{\underline{\underline{c}}}^{-1}\right){\underline{\underline{\underline{\underline{\Lambda }}}}}^{c}\left(\underline{\underline{I}}-{\underline{\underline{c}}}^{-1}\right)=\frac{G\left(\phi_{a}\right)}{2\tau_{R}(\mathrm{tr}\underline{\underline{c}},\phi_{a})}\left(\mathrm{tr}\underline{\underline{c}}-6+\mathrm{tr}{\underline{\underline{c}}}^{-1}\right).$$

Because the conformation tensor is always a positive semi-definite, the resulting expression is always non-negative (the relaxation time is always positive). Similarly, the third and fourth terms, $\sigma_{s,M_{i}},i=0,2$, representing the entropy production due to moment relaxation, are also always non-negative for the choices made here for the corresponding transport coefficients. On the other hand, the fifth and sixth terms, $\sigma_{s,cM_{i}},i=0,2$, representing mixed elastic moment relaxation-induced entropy production terms, are in principle indeterminate signs. The only limitation that can be imposed from the thermodynamic consistency is that their contributions together with those of direct entropy production contributions due to relaxation elasticity, $\sigma_{s,c}$, and due to the corresponding moment relaxations, $\sigma_{s,M_{i}},i=0,2$, should each be non-negative

(43) $$\sigma_{s,t_{i}}\equiv \sigma_{s,c}+\sigma_{s,M_{i}}+\sigma_{s,cM_{i}}\geq 0,\;i=0,2.$$

**Figure 12:**
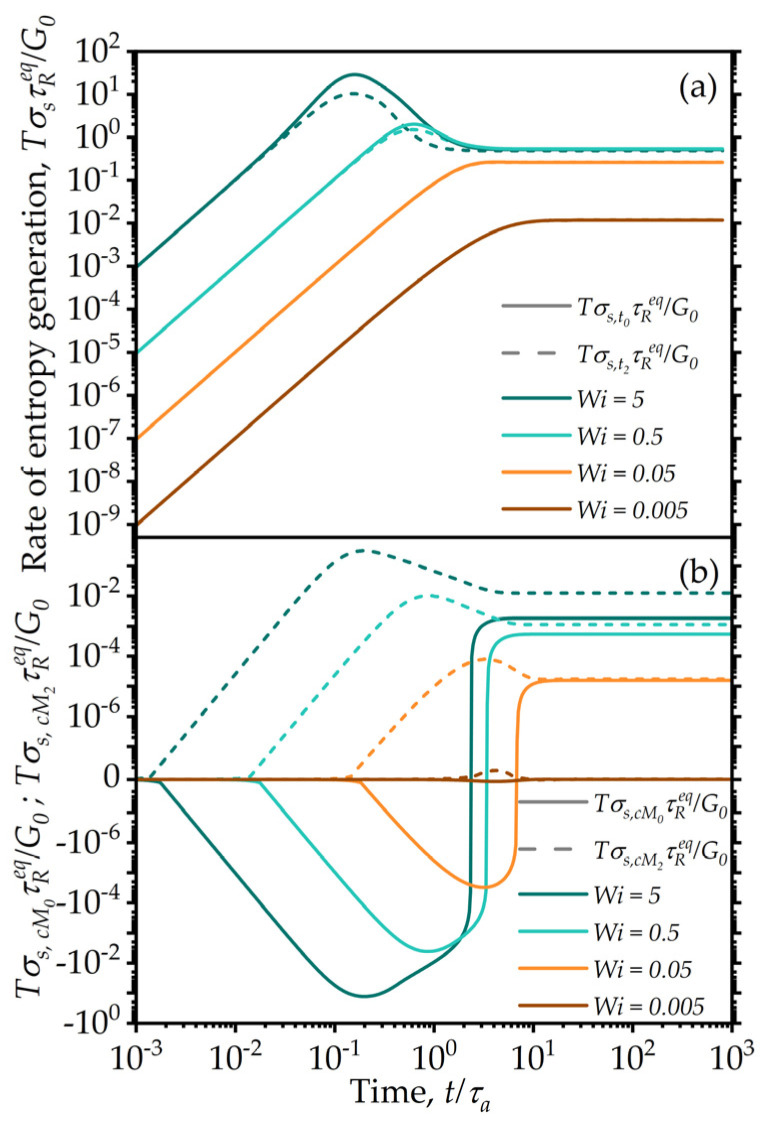
(**a**) Model predictions for the entropy production corresponding to the total relaxation terms, $T\sigma_{s,t_{i}},i=0,2$, in Equation (43) plotted for simple shear start-up from the quiescent state for various Weissenberg numbers. The different curves correspond to increasing Wi values, increasing from the bottom to top curves, with values as indicated in the insert legend. The contributions from the fifth and sixth terms in the entropy production (mixing terms) are explicitly plotted (**b**). It is clear that these contributions are not always non-negative; however, their additive contribution to the total relaxation terms, $T\sigma_{s,t_{i}},i=0,2$, in Equation (43) is always positive. The model parameters are $\lambda_{ba}=100,\;\lambda_{Ra}=0.5,\;k=0$.

The numerical evidence shows that these terms are indeed non-negative for the choice of the transport coefficients made here in the start-up of shear flows for all Wi values used, as seen in Figure 12a. However, note that taken on their own, the mixing terms are not individually required to be non-negative (as seen in Figure 12b).

For a more general investigation of the non-negative character of the entropy production, applicable for general flows or more general expressions of the Hamiltonian function, the SGBF allows one to examine the primitive dissipation bracket, and in particular the character of the two matrices that define it, namely the coupling Volterra derivatives of the viscous tensor $\underline{\underline{\underline{\underline{Q}}}}$ with respect to odd variables (velocity gradients) and the super matrix coupling the Volterra derivatives of all even variables (in this case, the six independent components of the conformation tensor and the two moments), as shown in Equation (17). Note that odd and even variables (referring to symmetry upon time inversion) do not couple with each other (i.e., the Curie principle [80]); therefore, their effects can be examined separately. For non-negative entropy production, it suffices that the $\underline{\underline{\underline{\underline{Q}}}}$ tensor and super matrix are non-negative. It is easy to show that for the $\underline{\underline{\underline{\underline{Q}}}}$ tensor, due to the simplicity of the isotropic description proposed for it in Equation (31), the thermodynamic restrictions translate to a shear viscosity that is required to be non-negative. It is more difficult to show the super matrix coupling for all structural variables, as this must satisfy the necessary and sufficient condition of Sylvester’s criterion for positive semi-definiteness, where all possible principal minors must be non-negative. However, some necessary conditions can still be examined. For example, all diagonal terms (these are all non-negative, as one easily can see in the previous explanation) and some of the principal minors (denoted with ℳ) can be examined in an approximate fashion; we can show that the generic coupling of $\underset{=}{c}$ with either $M_{0}$ or $M_{2}$ after an order of magnitude analysis around the static equilibrium leads to:

(44) $$\begin{array}{l} {ℳ}_{c,M_{0}}^{eq}∼\left\|{\underline{\underline{\underline{\underline{\Lambda }}}}}^{\left(c\right)}\right\|\left\|\Lambda_{M_{0}}^{\left(a\right)}\right\|-{\left\|{\underset{=}{\Lambda }}_{M_{0}}^{\left(b\right)}\right\|}^{2}\\ {ℳ}_{c,M_{2}}^{eq}∼\left\|{\underline{\underline{\underline{\underline{\Lambda }}}}}^{\left(c\right)}\right\|\left\|\Lambda_{M_{2}}^{\left(a\right)}\right\|-{\left\|{\underset{=}{\Lambda }}_{M_{2}}^{\left(b\right)}\right\|}^{2} \end{array}$$

For the order of magnitude analysis, the elastic modulus can be assumed to be $G(\phi_{a})∼n_{p}k_{B}T$. After simplification from Equation (44), we get:

(45) $$\begin{array}{l} {ℳ}_{c,M_{0}}^{eq}\approx \frac{M_{0}^{2}}{\tau_{R}^{eq}\tau_{M_{0}}^{\left(a\right)}}-\frac{1}{{\left(\tau_{M_{0}}^{\left(b\right)}\right)}^{2}}\geq 0\Rightarrow \lambda_{ba}^{2}M_{0}^{2}\geq \lambda_{Ra}\\ {ℳ}_{c,M_{0}}^{eq}\approx \frac{M_{0}}{\tau_{R}^{eq}\tau_{M_{2}}^{\left(a\right)}}-\frac{1}{{\left(\tau_{M_{2}}^{\left(b\right)}\right)}^{2}}\geq 0\Rightarrow \lambda_{ba}^{2}M_{0}\geq \lambda_{Ra} \end{array}$$

Far from equilibrium, $M_{0}$ assumes a large value assuring that these constraints are satisfied. The parameter ranges selected for investigation in the previous sections obey these constraints at equilibrium and the corresponding numerical experiments indicate non-negative entropy generation.

**Figure 13:**
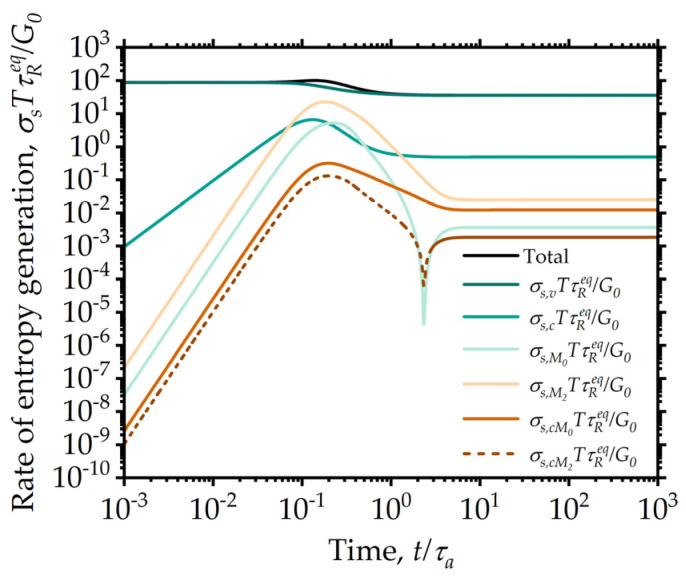
Model predictions for individual contributions to the entropy production, as indicated in Equation (41) for the simple shear start-up test for Wi=5. The dissipation is by far dominated by the viscous effects, whereas the contribution from the structural moments is smaller by several orders of magnitude. Negative values are indicated with a dashed line. Model parameters are: $\lambda_{ba}=100,\;\lambda_{Ra}=0.5,\;k=0$.

The entropy expression can also be used to judge the evolution and the importance of various dissipative phenomena during transients. The entropy production rates corresponding to the six terms in the Equation (41) are shown for a simple shear flow start-up case in Figure 13. Note that of all the contributions, only the fifth one is occasionally negative (indicated with a dashed line). However, as noted, the total entropy production still satisfies all thermodynamic requirements, ensuring that the resulting system of equations is thermodynamically consistent for the parameters chosen. It is also of interest to note that most of the entropy production is due to the viscous contribution.

## 5. Summary and Outlook

A thermodynamically consistent modeling approach was applied to a particle-level physical description of an aggregating suspension that systematically extracts the macroscopic rheological behavior from the underlying physics of the aggregation and breakage. The approach relates the entropy and free energy change involved in the evolution of agglomerate sizes with the rate equation of aggregation and breakage, which is in turn used to describe the evolution of elasticity, plasticity and thixotropy. The approach uses the SGBF-NET developed by Beris and Edwards [43] and the governing equations for suitable transport coefficients such as those suggested here, which are thermodynamically consistent, i.e., they obey the first and the second law of thermodynamics and follow additional constraints given by Onsager–Casimir reciprocal relations. Importantly, the rate processes dependent upon the flow are explicitly dependent upon the conformational stress and not the shear rate, as is required for thermodynamic consistency, which sets this work apart from other thixotropy models, potentially leading to enhanced model stability.

The framework is applied to a simplified set of independent system variables for a suspension of agglomerates described by a lognormal distribution to generate a set of closed equations that describe the stress and agglomerate structure valid for arbitrary, noninertial flows. The resultant equations have a total of 10 parameters that can be related to the physical properties of the system. Although developed here based on a generic simplified physical picture for illustration purposes, these model equations can be readily extended to include more complex underlying physical processes beyond the aggregation and breakage processes considered here, as well as more complex distributions for any specific system of interest based on available relevant experimental data. Inversely, one can possibly simplify the present model by assuming a uniform distribution that only involves the zeroth-order moment of the distribution. This will lead to only one differential equation for the particle size distribution that may be simpler to solve; however, the important thing to note is that such simplicity may be at the expense of a more appropriate and physically meaningful description of the system.

The examples of model behavior for a highly simplified set of parameter values show the rich behavior in qualitative agreement with general trends and key signatures observed in thixotropic elasto-viscoplastic materials via experimentation. Another benefit of this approach is that because it uses physically achievable measures of structure (such as the agglomerate volume and fractal dimension), it allows one to incorporate independently derived structural models and relationships instead of relying on phenomenology. Moreover, it potentially allows for an independent verification of the structural modeling characteristics, as they are in principle experimentally testable. The functional forms and scaling in the rate equation can be directly paired with population balance models for thermodynamically consistent modeling of mesoscale aggregation and breakage dynamics [2,3]. This methodology also provides a clear path to incorporate nonhomogeneous effects, such as stress-induced migration, because the model uses the densities and concentrations of agglomerates that can be modeled to vary spatially.

There are additional phenomena such as dynamic arrest [81] and anisotropy [82] that become prominent in densely packed systems. The dimensionless agglomerate volume, $\phi_{a}$, introduced in this work can be related to spatial packing; however, capturing the exact nature of this phenomenon remains challenging to address from a thermodynamic perspective and is an issue worthy of further investigation in the future.

## Appendix A. Entropy of Lognormal Distribution

For a lognormally distributed quantity, the entropy is defined as:

(A1) h(x)=−E[lnf(x)],

where f(x) is the lognormal density function:

(A2) $$f(x)=\frac{1}{x\sigma \sqrt{2\pi }}\exp\left[-\frac{{\left(\ln x-\ln x_{0}\right)}^{2}}{2\sigma^{2}}\right].$$

This equation can be rewritten in term of the standard normal variable Z using the transformation, $X=\exp\left(\ln x_{0}+\sigma Z\right)$:

(A3) $$f(X)=\frac{1}{X\sigma \sqrt{2\pi }}\exp\left(-\frac{Z^{2}}{2}\right),$$

where Z is the standard normal variable. Substituting Equation (A3) in the definition of the entropy given by (A1), we get:

$$\begin{array}{cc} h(x) & =-E\left[\ln f(x)\right]\\ & =E\left[\frac{Z^{2}}{2}+\ln\left(\sigma \sqrt{2\pi }\right)+\ln X\right]\\ h(x) & =E\left[\frac{Z^{2}}{2}+\frac{1}{2}\ln\left(2\pi \sigma^{2}\right)+\ln x_{0}+\sigma Z\right]. \end{array}$$

Because E[Z]=0 and $E\left[Z^{2}\right]=0$:

(A4) $$h(x)=\frac{1}{2}+\frac{1}{2}\ln\left(2\pi \sigma^{2}\right)+\ln x_{0}.$$

## Appendix B. Volterra Functional Derivatives

An arbitrary functional F in the bracket formula dependent on a continuous function *w* can be expressed as:

(A5) $$F[w]=\int_{\Omega }f(w,\nabla w,\nabla^{2}w,\ldots )\;dV.$$

With the formal definition of the Volterra functional derivative being:

(A6) $$\underset{\delta w\to 0}{\lim}\left(F[w+\delta w]-F[w]\right)=\int_{\Omega }\frac{\delta F}{\delta w}\;\delta w\;dV,$$

the Volterra functional derivative can be obtained as:

(A7) $$\frac{\delta F}{\delta w}=\frac{\partial f}{\partial w}-\nabla_{\alpha }\left[\frac{\partial f}{\partial \left(\nabla_{\alpha }w\right)}\right]+\nabla_{\alpha }\nabla_{\beta }\left[\frac{\partial f}{\partial \left(\nabla_{\alpha }\nabla_{\beta }w\right)}\right]\cdots .$$

This definition needs to be trivially modified if the variable function is a non-scalar tensor. Moreover, if there are constraints imposed on the functions (as in the case of a divergence-free momentum density for an incompressible fluid), the Volterra derivative has to also satisfy the same constraints—see [43] for more details. Taking everything into account, the Volterra derivatives of the Hamiltonian with respect to the system state variables can be easily calculated for the chosen state variables in the text and the Hamiltonian function as:

(A8) $$\frac{\delta H}{\delta m_{\alpha }}=\frac{m_{\alpha }}{\rho }\equiv v_{\alpha },$$

(A9) $$\frac{\delta H}{\delta M_{0}}=-\frac{n_{p}k_{B}T}{2}\left(-2-2\ln M_{0}+\ln\left(\frac{2\pi \sigma^{2}}{\exp\sigma^{2}}\right)+\frac{1}{\sigma^{2}}\right)+\frac{\partial G(\phi_{a})}{\partial M_{0}}\left(\mathrm{tr}\left(\underset{=}{c}-\underset{=}{I}\right)-\ln\det\underline{\underline{c}}\right),$$

(A10) $$\frac{\delta H}{\delta M_{2}}=-\frac{n_{p}k_{B}T}{2}\frac{M_{0}\left(1-\sigma^{2}\right)}{M_{2}\sigma^{2}}+\frac{\partial G(\phi_{a})}{\partial M_{2}}\left(\mathrm{tr}\left(\underset{=}{c}-\underset{=}{I}\right)-\ln\det\underset{=}{c}\right),$$

(A11) $$\frac{\delta H}{\delta c_{\alpha \beta }}=\frac{G(\phi_{a})}{2}\left(\delta_{\alpha \beta }-c_{\alpha \beta }^{-1}\right).$$
